# Stimuli-responsive nanoplatforms for sonodynamic immunotherapy: opportunities and challenges in cancer treatment

**DOI:** 10.3389/fimmu.2026.1845672

**Published:** 2026-05-29

**Authors:** Chunyu Zhang, Shu Bu, Xiaoyu Wang, Xin Shen, Yang Du, Jiangnan Yang, Zhiwei Miao

**Affiliations:** 1 Department of Gastroenterology, Zhangjiagang Traditional Chinese Medicine (TCM) Hospital Affiliated to Nanjing University of Chinese Medicine, Zhangjiagang, China; 2 First Clinical Medical College, Nanjing University of Chinese Medicine, Nanjing, China; 3 Department of General Surgery, Clinical Medical College, Yangzhou University., Yangzhou, China; 4 Key Laboratory of Translational Cancer Stem Cell Research, Department of Pathophysiology, Hunan Normal University Health Science Center, Changsha, China

**Keywords:** cancer immunotherapy, immunogenic cell death, nanomedicine, sonodynamic therapy, stimulus-responsive

## Abstract

Stimulus-responsive sonodynamic immunotherapy is a promising non-invasive strategy that combines ultrasound-activated Sonodynamic therapy (SDT) with a smart stimulus-responsive nanodelivery system to overcome the limitations of traditional cancer therapies. By utilizing signals from the tumor microenvironment (TME), such as acidic pH, high glutathione (GSH) levels, enzyme overexpression, and hypoxia, stimulus-responsive nanosonic sensitizers enable precise, on-demand release and deep tumor penetration. Ultrasound activation generates reactive oxygen species (ROS) and cavitation effects, inducing immunogenic cell death (ICD), characterized by damage-associated molecular patterns (DAMPs) and exposure/release of tumor-associated antigens (TAAs). This approach can transform “cold” tumors into “hot” tumors, promoting dendritic cell maturation, cytotoxic T lymphocyte (CTL) infiltration, and systemic antitumor immunity, including remote effects and long-term immune memory. When combined with immune checkpoint blockade (ICB), chemodynamic therapy (CDT), gas therapy, photothermal (PTT)/photodynamic therapy (PDT), or gene therapy, stimulus-responsive sonodynamic immunotherapy can significantly improve tumor suppression rates, reduce metastasis, and minimize systemic toxicity. Despite significant progress in preclinical studies, clinical translation still faces numerous challenges, including low ICD efficiency in hypoxic tumors, TME heterogeneity, parameter standardization, and optimization of the dual role of High migration group box 1 (HMGB1). This review summarizes the mechanisms, stimulus-response strategies, multimodal synergistic effects, and current clinical progress of stimulus-responsive sonodynamic immunotherapy, highlighting its opportunities and key challenges in future precision cancer treatment.

## 1 Introduction

Cancer has become one of the leading causes of death in the 21st century and a major global public health threat. According to the latest statistics from the World Health Organization, there were approximately 20 million new cancer cases and nearly 10 million deaths worldwide in 2022. Furthermore, it is projected that by 2050, the number of new cases will reach 35 million, an increase of 77%. The majority of the burden falls on low- and middle-income countries (1). Breast cancer has surpassed lung cancer to become the most common type of cancer, while colorectal cancer, liver cancer and cervical cancer are prevalent in specific regions, reflecting the geographical and economic heterogeneity of the cancer spectrum. Traditional treatments, such as surgery, chemotherapy and radiotherapy, have been effective in local control and short-term survival improvement, but their inherent limitations are becoming increasingly prominent: surgery is difficult to eradicate micrometastases; chemotherapy lacks tumor selectivity, leading to serious systemic toxicity; radiotherapy can be precisely targeted, but is limited by the dose tolerated by normal tissues and has limited efficacy in hypoxic tumor core areas (2). In recent years, immunotherapy, especially ICB (such as anti-PD-1/PD-L1, anti-CTLA-4 antibodies), adoptive cell therapy (ACT, such as CAR-T cell therapy) and tumor vaccines (including mRNA vaccines, dendritic cell vaccines) have brought revolutionary breakthroughs to cancer treatment, and some patients have achieved long-term remission or even cure. However, its overall response rate is still low, mainly limited by the following key mechanisms: 1. Immunosuppression mediated by TME: TME is composed of tumor cells, stromal cells, immunosuppressive cells (such as myeloid-derived suppressor cells (MDSCs), regulatory T cells (Tregs), M2 macrophages) and extracellular matrix, forming physical barriers (high interstitial pressure, dense fibrosis) and chemical barriers (low pH, high lactate, hypoxia, GSH over expression). These factors work together to inhibit the infiltration and function of effector T cells (Teff), maintaining an “immune desert” or “cold tumor” state. For example, immunosuppressive factors such as TGF-β, IL-10 and VEGF significantly weaken the maturation of DCs and the efficiency of antigen presentation (3). 2. Intratumoral heterogeneity: This includes genomic instability (high tumor mutational burden (TMB) vs. low TMB), epigenetic variations, and clonal evolution, leading to significant differences in the sensitivity of different subpopulations within the same tumor to the same therapy. ICB efficacy is highly dependent on neoantigen load, but most solid tumors have low neoantigen expression, limiting T cell recognition and killing (4). 3. Immune-related adverse events: ICBs trigger autoimmune responses by relieving T cell suppression. Common immune-related adverse events (irAEs) include rash, colitis, pneumonia, hepatitis, and endocrine disorders. The incidence of severe (grade 3–4) irAEs reaches 10%–20%, and some require discontinuation of treatment. Adoptive Cell Transfer Therapies (ACTs) can induce cytokine release syndrome (CRS) and neurotoxicity, limiting their application in solid tumors (5).

The above-mentioned multiple bottlenecks together constitute the “drug resistance triangle” of current cancer treatment - insufficient local control, systemic immune escape and treatment-related toxicity, and there is an urgent need to develop new strategies that can deeply penetrate the TME, accurately activate endogenous immunity and have a good safety window. As an ultrasound-driven *in situ* activation therapy, SDT provides a new path to break through the limitations of traditional and immunotherapy with its non-invasiveness, deep penetration ability and controllable ICD induction characteristics (6).

## 2 SDT-based ICD induction potential and stimulus-response strategies

### 2.1 Advantages of SDT

SDT is an emerging non-invasive tumor treatment strategy. Its core is to use focused ultrasound (FUS) as an energy source to remotely activate sonosensitizers enriched in the tumor, and generate ROS, acoustic cavitation and local thermal effects in the designated area to achieve highly selective killing of tumor cells (7). Unlike traditional PDT which relies on visible/near-infrared light (penetration depth <1 cm), the low-intensity ultrasound (frequency 0.5–3 MHz, intensity 1–3 W/cm²) used in SDT has excellent tissue penetration ability (>10 cm) and can reach deep solid tumors (such as liver cancer, pancreatic cancer and glioma) without attenuation, breaking through the bottleneck of phototherapy in the treatment of deep tumors (8).

The treatment process of SDT is divided into three key steps:”targeted enrichment - ultrasound activation - multimodal killing” (9). First, through the Enhanced Permeability and Retention (EPR) effect or active targeted modification (such as cRGD, folic acid, antibody), the sonosensitive agent (such as heme chloride Ce6, IR780, protoporphyrin IX (PpIX), hematoporphyrin monomethyl ether HMME, 5-aminolevulinic acid (5-ALA)) is selectively accumulated in the tumor tissue (10). Then, *in vitro* ultrasound is focused on the tumor area to stimulate the sonosensitive agent to jump from the ground state to the excited state, and convert the surrounding oxygen molecules into highly cytotoxic singlet oxygen (^1^O_2_), superoxide anion (·O_2_^-^), hydroxyl radical (·OH) and other ROS through electron/energy transfer (11). At the same time, the ultrasound-induced cavitation effect (including steady-state cavitation and inertial cavitation) generates microjets, shock waves and sonoluminescence, which further amplify mechanical cell membrane damage and mitochondrial endoplasmic reticulum stress (12).

SDT’s multi-effect synergistic killing mechanism gives it significant tumor selectivity: ① ROS-mediated oxidative stress: directly attacks DNA, lipids and proteins, leading to irreversible apoptosis, necrosis or ferroptosis; ② Cavitation effect: destroys the tumor vascular endothelium and matrix barrier, enhancing drug penetration; ③ Local thermal effect: local temperature is less than 43 °C, avoiding non-specific burns from traditional thermotherapy, while synergistically amplifying mitochondrial damage with ROS (13). Compared with radiotherapy (requiring high dose of ionizing radiation) and chemotherapy (systemic toxicity), SDT produces almost no ROS and cavitation damage in normal tissues, has a wide safety window, and can be activated in a “spatiotemporally controllable” manner by adjusting ultrasound parameters (14).

### 2.2 The potential of SDT to induce ICD

**Figure 1:**
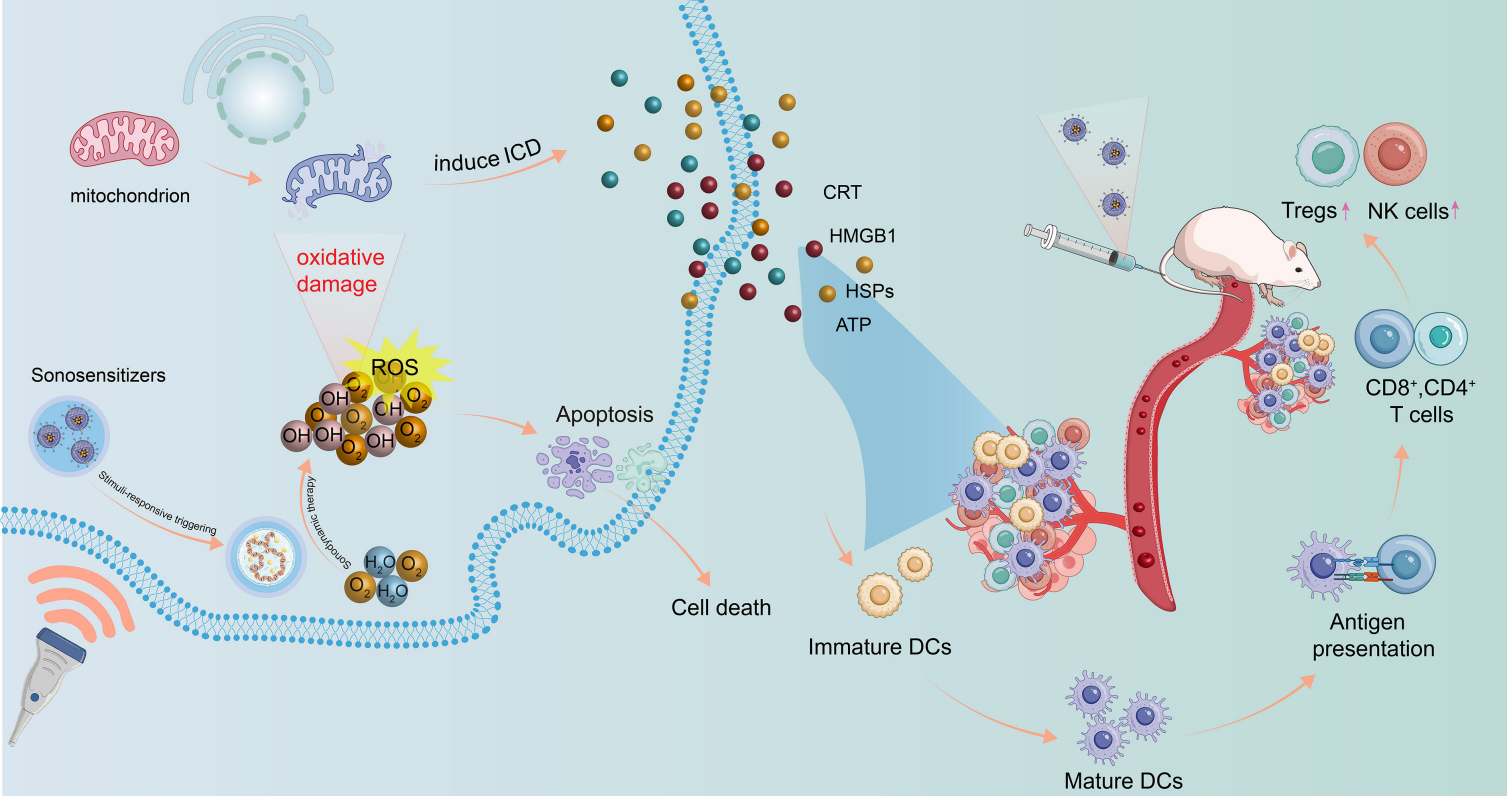
The core mechanism and process of SDT-induced ICD.

SDT uses ultrasound to activate a sonosensitive agent to generate high levels of ROS and cavitation mechanical stress *in situ*. This not only directly kills tumor cells but also induces ICD, transforming the tumor into an “*in situ* vaccine.” It releases key DAMPs and TAAs, systematically activating dendritic cell (DC) maturation, antigen cross-presentation, and CTL infiltration, thereby reversing the immune “cold” tumor state to a “hot” tumor state and significantly enhancing the anti-tumor immune response (15). SDT-induced ICD is centered on ROS-mediated endoplasmic reticulum stress and mitochondrial damage, triggering a series of characteristic DAMPs exposure and secretion:① Calreticulin (CRT): rapidly translocates from the endoplasmic reticulum to the cell membrane surface, acting as a “phagocytosis” signal to promote the phagocytosis of apoptotic tumor cells by DCs, and significantly upregulates the expression of CD80/CD86 co-stimulatory molecules; ② HMGB1 is passively released from the cell nucleus, and combines with TLR4/MyD88 signal to activate DCs to produce IL-1β and TNF-α, enhancing antigen presentation efficiency; ③ Adenosine Triphosphate(ATP), as a “find me” signal, recruits DCs and neutrophils to the tumor bed through the P2X7 receptor; ④ Heat shock proteins (HSP70/HSP90) are exposed on the cell surface, assisting antigen peptides in loading into MHC-I/II molecules, and enhancing the activation of CD8^+^ and CD4^+^ T cells (16). Meanwhile, SDT-induced necrotizing lysis releases a large number of TAAs, forming a rich neoantigen library and overcoming the limitations of low tumor immunogenicity (17). **Figure 1** illustrates the core mechanism and process of SDT-induced ICD.

*In vivo* studies have confirmed that in SDT-treated 4T1 breast cancer, CT26 colon cancer, and B16F10 melanoma models, CRT exposure can be increased by 5–10 times, HMGB1/ATP secretion increases by 3–8 times, accompanied by a significant increase in the proportion of DCs maturation markers (CD11c^+^MHC-II^+^), a 4–6-fold increase in the CD8^+^ T cell proliferation index in tumor draining lymph nodes (TDLN), and a reversal of the intratumoral CD8^+^/Treg ratio to >2 (18). More importantly, SDT-induced ICD can elicit an abscopal effect: in bilateral tumor models, unilateral SDT treatment not only inhibited the growth of the tumor *in situ*, but also significantly inhibited the unirradiated distal tumor, accompanied by increased CD8^+^ T cell infiltration and upregulation of IFN-γ secretion in the distal tumor, confirming the establishment of systemic immune memory (19).

Compared to ICD induced by traditional chemotherapy (such as oxaliplatin), SDT has unique advantages: ① No systemic activation is required, avoiding systemic inflammatory storm; ② Ultrasound is precisely focused, and the ICD is confined to the tumor area, reducing irAEs; ③ Cavitation effect disrupts the physical barrier of TME, promoting antigen release and immune cell penetration; ④ It can be combined with ICBs (such as anti-PD-L1) to increase the response rate from <20% to >60% (20). Preclinical data show that the SDT-ICD strategy can still effectively activate the immune cycle in low TMB, MSI stable “cold” tumors (such as pancreatic cancer and glioma), break through the bottleneck of ICB monotherapy resistance, and provide a new paradigm for immunotherapy of refractory tumors (21–25). **Figure 1** illustrates the complete mechanism by which SDT activates a sonosensitive agent to produce ROS, induces ICD in tumor cells and releases DAMPs, thereby promoting dendritic cell maturation, antigen presentation and activation of CD8^+^/CD4^+^ T cells and NK cells, thus triggering a systemic anti-tumor immune response.

The choice of administration route for sonosensitizers significantly influences their biodistribution, off-target exposure, and downstream immunological outcomes. Systemic intravenous administration remains the most common route due to its convenience and ability to leverage the EPR effect for passive tumor accumulation. However, it may lead to relatively higher off-target distribution in reticuloendothelial system organs (liver, spleen) and potential transient photosensitivity or oxidative stress in normal tissues. In contrast, local intratumoral injection can achieve higher local drug concentration and lower systemic exposure, but is limited by poor distribution uniformity in large or deep-seated tumors and technical challenges in clinical translation. Stimulus-responsive nanoplatforms developed in recent years can effectively improve the therapeutic index regardless of administration route. By responding to TME cues (pH, GSH, enzymes, hypoxia), these smart carriers promote deep tumor penetration, reduce premature release in circulation, and enhance tumor-specific accumulation. Consequently, systemic administration combined with stimulus-responsive design and focused ultrasound activation can achieve favorable tumor-to-normal tissue ratios, minimize off-target ROS generation, and ultimately augment ICD efficiency while preserving systemic immune homeostasis (26). Future optimization may involve biomimetic or active targeting strategies to further refine biodistribution and potentiate abscopal immune effects.

### 2.3 The necessity of stimulus-response release strategy in SDT precision delivery and immune synergy

TME is highly heterogeneous and dynamic. Its characteristic signals—acidic pH (6.5–6.8), high expression of matrix metalloproteinases (MMPs)/cathepsin B (CTSB), redox imbalance, and chronic hypoxia (pO2 <5 mmHg)—provide an endogenous “trigger” for designing intelligent stimulus-responsive nanodelivery systems. The stimulus-response release strategy, by responding to the above TME specific signals, realizes the “on-demand” precise release of sonosensitive agents and deep penetration into the tumor, significantly improving tumor accumulation, ROS localization and ICD efficiency. It is the core enabling technology for SDT to leap from “cell killing” to “immune activation” (26). Specific stimulus-response release strategies include: pH response, redox response, enzyme response, hypoxia response and multiple responses. pH response: The acidity of the tumor extracellular fluid is due to the Warburg effect and proton pump overexpression. pH-sensitive bonds (such as Schiff bases, acetals, and hydrazone bonds) break rapidly in acidic TME, triggering the depolymerization of nanocarriers and the burst release of sonic sensitizers (27). Redox response: The concentration of GSH in tumor cells is 100–1000 times that of normal tissues, which can reduce disulfide bonds (–S–S–) or selenium bonds. The ROS/GSH dual-response system achieves “adaptive” cascade amplification: for example, AMNP@J+C breaks down –S–S– under the ROS triggering generated by SDT, releasing the BRD4 inhibitor JQ1 to downregulate PD-L1, while MnFe2O4 consumes GSH to amplify the 1O2 yield, and in conjunction with ICB, increases the tumor inhibition rate from 45% to 92% (28). Enzyme response: MMP-2/9, CTSB, and γ-glutamyl transpeptidase (GGT) are overexpressed in TME. Enzyme-cleaved peptides (such as GPLGVRG, GFLG) or polymers (such as PEGylated polylysine) are specifically hydrolyzed, exposing the target ligand or releasing the sonosensitive agent. For example, EIPS nanoparticles remove the PEG shell under CTSB catalysis, inhibit exosomal PD-L1 secretion, restore CTL killing function, and the distant tumor inhibition rate in the CT26 model reaches 68% (29). Hypoxia response: Hypoxia induces HIF-1α upregulation, activates the reduction of hypoxia-sensitive groups such as nitroimidazole/azobenzene. MoOx-PEG nanoparticles are reduced to Mo⁵^+^in the hypoxia region, activate the STING pathway to produce type I interferon, and at the same time catalyze H_2_O_2_ to O_2_ to improve SDT efficiency. In the B16F10 model, IFN-β secretion increased by 5.7 times, and the proportion of systemic CD8^+^T memory cells increased to 28% (30). Multiple response: Single response is difficult to cope with the complexity of TME. Multiple response systems (such as pH/GSH/ROS triple lock) realize sequential release and signal amplification. PEG-PPMDT NPs are exposed to the cationic layer after PEG removal in acidic TME. After internalization, GSH breaks down –S–S– to release Ce6. The ROS generated by SDT further cleaves TiO_2_to produce ·OH, forming a “ROS storm”. The expression of ICD markers is upregulated by 8–12 times (31).

The necessity of stimulus-response strategy is reflected in: ① Enhancing tumor accumulation and penetration: After response, the particle size shrinks (<50 nm) and the surface charge flips to break through the physical barrier of TME (32); ② Reducing off-target activation: Maintaining stability in normal tissues at pH 7.4 and GSH <10 μM to avoid systemic toxicity (33); ③ Synergistic immune reprogramming: Releasing immune adjuvants (such as CpG, JQ1) or oxygen-consuming regulators, synergistically transforming “cold” tumors into “hot” tumors with ICD, and achieving >90% tumor clearance and anti-relapse immunity in combination with ICB (34). Preclinical data show that stimulus-response SDT can increase the objective response rate (ORR) from 15% with ICB monotherapy to 67% in PDX models, providing a translatable pathway for precision immunotherapy (35–39).

## 3 Basic principles of sonodynamic immunotherapy

### 3.1 Core mechanism of SDT-induced ICD

The core mechanism of SDT inducing ICD starts with ultrasound (US) activating sonosensitive agents to generate high levels of ROS and sonocavitation effect *in situ*. Through multiple death pathways, it synergistically damages tumor cells, triggers the release of characteristic DAMPs and TAAs, and ultimately drives the maturation of dendritic cells (DCs), antigen cross-presentation and activation of CTLs, forming a complete “tumor-immunity” positive feedback loop (40). **Table 1** summarizes the key quantitative conclusions of stimulus-responsive sonodynamic immunotherapy.

**Table 1:** Summary of key quantitative claims in stimulus-responsive sonodynamic immunotherapy.

Nanoplatform composition	Mechanism & context	Quantitative claim	Acoustic parameters	Dosing	Tumor model	Reference
Oxygen-deficient molybdenum oxide nanoparticles (MoO_x_-PEG)	Hypoxia-responsive; activates STING pathway	In cSDT group (MoO_x_-PEG + US + aCTLA-4): 3/5 primary tumors completely regressed; significant inhibition of distant tumors and lung metastases; markedly increased DC maturation, CD8^+^ T cell infiltration, and cytokine secretion (TNF-α, IL-6); prolonged mouse survival	40 kHz, 3 W cm^-^², 15 min (3 times)	MoO_x_-PEG: 20 mg kg^-1^ (i.v., days 0, 2, 4); aCTLA-4: 20 μg/mouse (i.v., days 1.5, 3.5, 5.5)	4T1 breast cancer model (Balb/c mice, bilateral tumor + lung metastasis models)	(30)
Ov-MO@CPO nanospheres (oxygen vacancy-rich MnO_2_)	pH-responsive oxygen generation	Ov-MO@CPO-PD + US: significant tumor growth inhibition and reduced lung metastases in 4T1 model; M1 macrophages↑, M2↓; CD4^+^/CD8^+^ T cells, NK cells, B cells infiltration↑; Tregs↓; elevated serum cytokines (IL-1β, IL-6, IL-12p70, TNF-α); combined with aPD-L1 further increases CD4^+^/CD8^+^ T cell infiltration and antitumor efficacy	1 MHz, 2 W cm^-^², 5 min (*in vivo*; 2 min *in vitro*)	10 mg kg^-1^ (i.v., every other day); aPD-L1: 20 mg kg^-1^ (i.v.)	4T1 breast cancer model (Balb/c mice, subcutaneous + lung metastasis)	(89)
PFP@PEG-CMD-Ce6 nanodroplets (phase-change)	Ultrasound cavitation enhancement	NB + US + aPD-L1 group: near-complete primary tumor regression (97.03% tumor weight inhibition); significantly increased CD8^+^ CTL infiltration; strong suppression of lung metastases; enhanced systemic antitumor immunity	Ultrasound irradiation (specific frequency/power/duration not detailed in main text; used for both *in vitro* and *in vivo* acoustic cavitation)	i.v. injection of NBs (therapeutic dose) + aPD-L1	RIPK3-deficient CT26 colon cancer syngeneic model (Balb/c mice, primary subcutaneous tumor + pulmonary metastasis)	(102)
LIP-PFH (perfluorohexane liposomes + HMME)	Cavitation-enhanced vascular disruption	Significant tumor growth inhibition and reduced lung metastases vs. control/nab-P; markedly ↑CD4^+^T/CD8^+^T cells in blood/spleen/tumor; ↓CD8^+^PD-1^+^T cells; ↑ICD markers and DC maturation; elevated IFN-γ	LIFU: 1 W/cm², 2 min (intratumoral activation, every 3 days)	Intratumoral multipoint injection (~10⁶ NPs in 100 µL), every 3 days	4T1 breast cancer model (Balb/c mice)	(103)
Mn-GMSs (MnWOx-loaded gelatin microspheres)	Embolization + gas + SDT	Significant tumor growth inhibition and reduced lung metastases in H22 mice (Mn-GMSs + US + αPD-L1 group: complete distant tumor regression in 3/5 mice); markedly ↑ mature DCs, CD8^+^ TILs, and cytokines (CXCL-10, IFN-β, IL-6, TNF-α); ↓ Tregs; strong efficacy in rat N1S1 and rabbit VX2 orthotopic liver tumor models	40 kHz, 3 W cm^-^², 5 min (mice, repeated 3 times); 10 min (rats/rabbits)	Intratumoral (mice: 200 μg MnWO_x_ in 2 mg GMSs) or intra-arterial embolization (rats: 300 μg MnWO_x_ in 3 mg GMSs; rabbits: 1 mg MnWO_x_ in 10 mg GMSs) + αPD-L1 (20 μg/mouse)	H22 subcutaneous (mice); orthotopic N1S1 (rats); orthotopic VX2 (rabbits)	(142)
PEG-CDM-aPD-L1/Ce6 (pH/ROS dual-responsive)	ICB co-delivery with SDT	P-aPD-L1/C + US: complete primary tumor inhibition; 83% mice survival >40 days; highest DC maturation (17.7%), CD8^+^ TILs (32.0%), IFN-γ^+^CD8^+^ T cells (54.5%); strong immune memory (TCM↑); negligible irAEs vs. free aPD-L1	2.0 MHz, 2.0 W cm^-^², 20% duty cycle, 5–10 min (applied 24 h post-injection)	Ce6 + aPD-L1: 5 mg kg^-1^ each (i.v., 5 times at 2-day intervals)	B16-F10 melanoma (C57BL/6 mice, subcutaneous)	(148)
Macrophage–cancer hybrid membrane-camouflaged nanoplatforms (ISZ@JUM): ICG and HIF-1α siRNA co-loaded ZIF-8 NPs (ISZ) coated with J774.A.1 macrophage/U87 GBM hybrid membrane (JUM)	Gene therapy + SDT	Significant tumor growth inhibition in orthotopic U87 model (ISZ@JUM + US group); effective HIF-1α mRNA/protein downregulation; enhanced intracellular ROS under hypoxia; good biosafety with no obvious toxicity or side effects	1.0 MHz, 40% duty cycle, 1.5 W cm^-^², 5 min (repeated 3 times)	10 mg kg^-1^ (i.v. injection)	Luciferase-expressing U87 orthotopic glioblastoma model (BALB/c nude mice)	(153)

#### 3.1.1 SDT-induced synergistic generation of ROS and cavitation effect

The synergistic generation of ROS and cavitation effect in SDT mainly relies on the activation of sonosensitizers (such as organic or inorganic compounds like Ce6, HMME, and IR780) by low-intensity focused ultrasound (typical parameters are 1–3 W/cm² power density and 0.5–2 MHz frequency) (41). Under the action of ultrasound, these sonosensitizers transition from the ground state (S₀) to the excited singlet state (^1^S*) through energy absorption, and subsequently undergo intersystem crossing (ISC) to a long-lived triplet state (^3^S*). In this triplet state, the sonosensitizer can interact with surrounding ground-state triplet oxygen (^3^O_2_) and other biological substrates through two primary photochemical pathways: ①Type I mechanism (electron transfer): The triplet-state sonosensitizer donates an electron to oxygen or other substrates, producing radical species such as ·O_2_^-^, which can further lead to the generation of ·OH and other ROS through subsequent cascade reactions. ②Type II mechanism (energy transfer): The triplet-state sonosensitizer transfers its energy directly to ground-state triplet oxygen (^3^O_2_), generating highly cytotoxic ^1^O_2_ (42). The ROS yield can reach 0.6–0.9, and can even be further improved in microbubble enhancement systems (43). At the same time, ultrasound also induces cavitation effects in endogenous or exogenous microbubbles in tissues, including stable cavitation and inertial cavitation. Cavitation, the violent expansion and collapse of bubbles, creates extreme local microenvironments on a microsecond scale, such as temperatures reaching thousands of K and pressures reaching hundreds of MPa. This leads to the dissociation of water molecules to generate ·OH and the ionization of oxygen molecules to generate ·O_2_^-^ and ^1^O_2_ (44). These cavitation processes generate high-speed microjets (velocities >400 m/s, reaching supersonic levels, directly shearing cell membranes and subcellular structures) and powerful shock waves (>100 m/s). MPa, propagating mechanical stress and sound energy, destroying the vascular endothelium and tumor microenvironment barrier) and sonoluminescence, thereby achieving the synergistic amplification effect of ROS generation and mechanical damage (45), ultimately significantly enhancing the damage to cell structures, such as causing mitochondrial membrane potential collapse and endoplasmic reticulum calcium ion leakage (46, 47).

#### 3.1.2 SDT-induced multi-pathway programmed/non-programmed cell death

SDT causes multiple programmed and non-programmed cell death pathways through triggered sonosensitive agents. The synergistic effects of these pathways lead to increased oxidative damage and immuneactivation which include apoptosis. ROS induce permeability of the mitochondrial outer membrane (MOMP) by oxidizing lipids of mitochondrial membranes. This results in the release of cytochrome c from the intermembrane space of mitochondria to the cytoplasm. This cytochrome c binds to Apaf-1 to form apoptotic bodies, thereby activating the caspase-9/3 cascade and promoting the cleavage of downstream substrates such as PARP, ultimately executing ordered cell disintegration. Simultaneously, the shear stress and mechanical disturbance generated by cavitation further activate cell surface death receptor pathways (such as Fas or TNF-R1), recruiting FADD and caspase. -8 initiates the extrinsic apoptosis pathway (48); secondly, necroptosis/pyroptosis occurs, in which high-intensity ROS excessively disrupts cell membrane integrity, leading to ion imbalance and cell swelling, activating the RIPK1/RIPK3-MLKL axis to form necrosomes and phosphorylating MLKL, causing it to oligomerize and form pores on the cell membrane, initiating programmed necrosis, or activating the NLRP3 inflammasome complex through oxidation, thereby cleaving gasdermin D (GSDMD) to produce N-terminal fragments, assembling pores on the membrane to release pro-inflammatory factors such as IL-1β and IL-18, leading to inflammatory cell lysis (49); In addition, ferroptosis is also one of the non-programmed cell death pathways. This is a form of iron-dependent death mediated by sonosensitive lipid peroxidation (LPO), which reduces GSH synthesis and depletes GPX4 enzyme activity by inhibiting the systemic Xc-transporter, leading to the accumulation of phospholipid peroxides that cannot be reduced. Simultaneously, Fe²^+^ions catalyze the Fenton reaction in the tumor microenvironment, generating a large number of ·OH, further amplifying lipid damage. The usual indicators are the enhancement of long-chain acyl-CoA synthase 4 (ACSL4) that encourages polyunsaturated fatty acid (PUFA) integration. The build-up of 4-hydroxynonenal (4-HNE) is terminal structure due to lipid peroxidation (50). To sum up, ultrasound (US) exposure of copper-based sonosensitive agents (e.g. Cu-TCPP) triggers copper ion Cu^+^release within cells, resulting in their death. The salt ions preferentially bind to lipoxygenated proteins in the mitochondrial respiratory chain complex leading to protein toxicity stress and tricarboxylic acid (TCA) cycle disorder. In addition, copper-dependent protein aggregation hinders iron-sulfur cluster assembly and respiratory enzyme activity, leading to non-apoptotic cell death (51, 52). Apoptosis/necrosis/ferroptosis ratios *in vivo* studies within 24 h after SDT were 35% (apoptosis), 28% (necrosis), and 22% (ferroptosis), which were all significantly higher than ROS alone (53).

#### 3.1.3 SDT-induced DAMPs/TAAs release and immune signaling cascade

The release of DAMPs and TAAs constitutes the pivotal link between SDT-induced immunogenic cell death and systemic antitumor immunity. ROS-induced endoplasmic reticulum stress leads to eIF2α phosphorylation and PP1 inhibition, driving rapid CRT translocation to the cell surface within 30 minutes as an “eat-me” signal (54). ATP is quickly released from the ER lumen (exposure >70%) and acts via P2X7 and LRP1 receptors to recruit and activate DCs. HMGB1 is passively released after nuclear membrane rupture, peaking at approximately 48 h, and engages TLR2/4 and RAGE to activate NF-κB signaling, thereby promoting pro-inflammatory cytokine secretion and T cell polarization (55, 56). Surface-exposed HSP70/HSP90 further serve as chaperones that facilitate antigen cross-presentation on MHC-I/II molecules (57). Necrotic lysis simultaneously liberates a broad repertoire of TAAs, increasing neoantigen diversity (58, 59).

This DAMPs/TAAs release initiates a robust DC-CTL immune activation cascade. Within 6–12 h post-SDT, mature DCs (CD11c^+^MHC-II^+^) in tumor-draining lymph nodes increase markedly, with CD80/CD86 expression upregulated approximately 3.8-fold (60). Subsequently, CD8^+^ T cell proliferation index reaches ~28% (Ki67^+^), the intratumoral CD8^+^/Treg ratio reverses to >3.2, and IFN-γ/granzyme B secretion rises 5–7 fold (61). In bilateral tumor models, these changes translate into significant abscopal effects, with distant tumor inhibition rates of ~62% and memory CD8^+^ T cells (T_EMRA) increasing to ~31%, establishing long-term immune memory (62–64).

SDT-induced ICD has been rigorously validated through multiple approaches. Flow cytometry typically shows CRT^+^Annexin-V^+^ cells exceeding 65% (65, 66). *In vivo* vaccination experiments demonstrated that SDT-treated tumor cells provided 100% protection against subsequent tumor re-challenge (67). Western blotting and ELISA confirmed strong activation of the cGAS-STING pathway (upregulation of cGAS, STING, and pIRF3), linking SDT-mediated DNA damage to type I IFN production and enhanced DC maturation and CD8^+^ T cell infiltration (68). These quantitative results not only substantiate the mechanistic framework but also highlight that ultrasound parameters and sonosensitizer dosage remain optimizable (69).

In summary, through synergistic ROS generation and cavitation effects, SDT drives multi-pathway cell death and orchestrated DAMPs/TAAs release, forming a complete mechanistic chain from localized tumor destruction to systemic immune activation, thereby providing a strong foundation for “cold” tumor immunotherapy (70).

### 3.2 Synergistic classification of SDT and immunotherapy

SDT is an amplification platform for a range of immunotherapies by inducing ICD to liberate DAMPs and TAAs. SDT, in conjunction with ICB, ACT, cytokine therapy, STING agonists, and novel immunotherapies, can systematically reshape TME, significantly enhancing effector T cell infiltration, functional activation, and immune memory formation. In 4T1 breast cancer, CT26 colon cancer, and B16F10 melanoma models, SDT-immunotherapy combination therapy increased tumor suppression rates from 30%–50% with single-agent therapy to 80%–95%, and effectively inhibited distant metastasis (71). The synergistic mechanism of SDT and ICB lies in ICD-mediated antigen release and PD-L1 expression inhibition: ROS generated by SDT oxidizes HIF-1α and downregulates PD-L1 transcription, while CRT/HMGB1 promotes DC maturation and recruits CD8^+^T cells for infiltration, significantly enhancing the response rate against PD-1/PD-L1 or against CTLA-4 (72, 73). SDT and ACT synergistically enhance tumor recognition and persistence of TILs or CAR-T by pre-treating tumors: TILs reinfused after SDT showed a 3.8-fold upregulation of IFN-γ secretion, and combined with HER2-CAR-T, tumor volume was reduced by 87% in solid tumor models, and the risk of CRS was reduced (74).

Cytokine therapy combined with SDT amplifies the inflammatory cascade: SDT-induced DAMPs activate NF-κB and upregulate IL-12/IFN-γ expression; low-dose IL-2 or GM-CSF combined with SDT can increase the maturation rate of DCs to 80%, and NK cell recruitment increased 4-fold in the B16F10 model, with a tumor clearance rate >90% (75). STING agonists and SDT are highly complementary: SDT damages mtDNA and activates cGAS-STING. MoOx-PEG nanoparticles loaded with STING agonists cascade under ultrasound to produce type I IFN (>12-fold), inhibiting MDSCs and enhancing T cell polarization. Combined with ICB, the distant tumor inhibition rate reached 68% in a bilateral tumor model (76). Novel therapies such as mRNA vaccines or CpG/R837 adjuvants further extend the synergy: SDT-ICD provides the antigen source, and the vaccine amplifies the clone to achieve personalized immune memory (77).

HMGB1 is a key DAMP for SDT induction, has a dual role: when promoting immunity (<300 ng/mL), HMGB1 binds to TLR4 to activate NF-κB, producing IL-1β/TNF-α, promoting DC maturation and T cell activation (IFN-γ^+^CD8^+^T ratio increases 3.5-fold); when promoting metastasis (>500 ng/mL), HMGB1 mediates platelet-TLR4 interaction, enhancing tumor cell adhesion and EMT. In the 4T1 model, lung metastasis was reduced by 65% in the HMGB1 knockout group (78). Therefore, it is necessary to accurately capture HMGB1 using biomimetic decoys (such as Lipo-Ce6/TPZ@MH) to block metastasis-promoting signals while preserving immune activation.

## 4 Classification and application of stimulus-response release strategies in SDT-induced ICD

**Figure 2:**
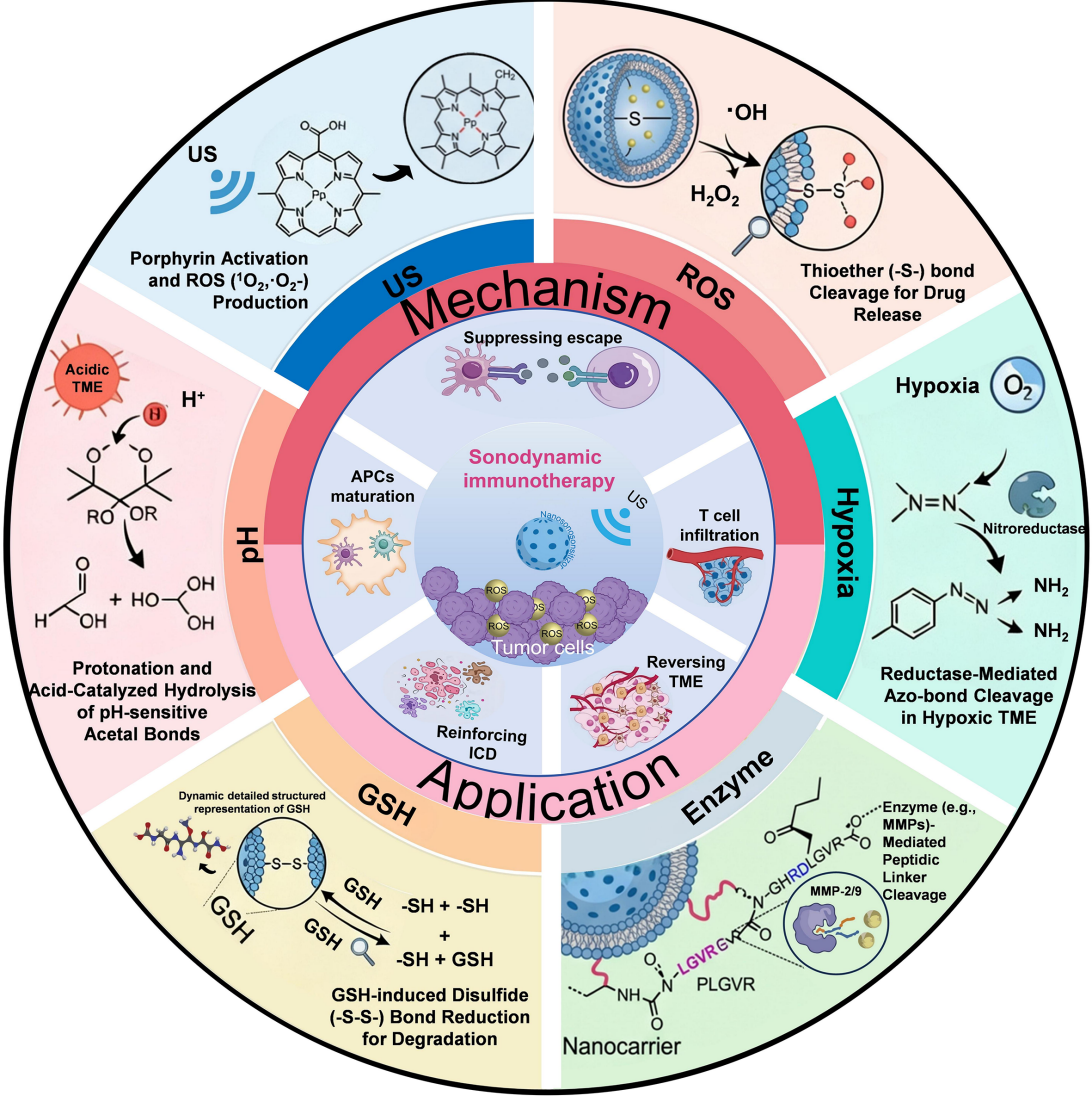
The application of responsive nanomaterials in tumor sonodynamic immunotherapy.

Strategies involving stimulus-response release are platforms or delivery technologies that utilize physiological signals unique to the TME as smart switches. Such platforms enable the activation and release of sonosensitive agents at the tumor site on demand. These technologies can significantly enhance the efficiency of SDT-induced ICD. In addition, they are capable of overcoming TME barriers such as hypoxia, acidity, high GSH, and over-expression of enzymes (79).These strategies are broadly classified into endogenous and exogenous categories based on their triggering signals. Endogenous single-response strategies, in particular, rely heavily on the intrinsic characteristics of the TME (such as pH gradients, redox states, enzyme activity, and hypoxic conditions). They achieve structural rearrangement, depolymerization, or drug release of nanocarriers by designing acid-sensitive bonds (such as hydrazone bonds and acetal bonds), reduction-sensitive bonds (such as disulfide bonds), enzymatically cleaved peptides (such as GPLGVRG), or hypoxia-reducing groups (such as nitroimidazole) (80). In various animal models such as 4T1 breast cancer, CT26 colon cancer, U87 glioma and PAN02 pancreatic cancer models, this strategy can increase the tumor accumulation rate of sonosensitive agents from the traditional 5%-10% ID/g to 15%-25% ID/g, upregulate the expression of ICD markers (such as CRT exposure, HMGB1 and ATP secretion) by 3–8 times, and promote DC maturation, T cell infiltration and the formation of long-term immune memory (81). This strategy not only reduces non-specific damage to normal tissues, but also lays the foundation for multimodal combination therapy (such as SDT combined with immune checkpoint inhibitors) and has high clinical translational potential (82, 83). **Table 2** summarizes the application of stimulus-responsive nano-sound sensory agents in SDT. See **Figure 2** for details, which illustrates the application of responsive nanomaterials in tumor sonodynamic immunotherapy.

**Table 2:** Stimuli-responsive nanosonosensitizers for SDT: classification, systems, and applications.

Classification	Type of response	Example materials/systems	Sonosensitizer release	Therapeutic approach	Triggering condition	Tumor model	Advantages	Limitations	Reference
Single Endogenous Response	pH-Responsive	Ov-MO@CPO-PD	O_v_-MO (oxygen vacancy-rich MnO_2_)	SDT + Immunotherapy	Acidic tumor microenvironment pH	Triple-negative breast cancer mouse model	Simple design; exploits natural tumor acidity for targeted activation; good biocompatibility	Limited to tumors with sufficient pH gradient; may have off-target effects in other acidic environments	(89)
Single Endogenous Response	Redox/GSH-Responsive	THPP-Oxa(IV)-PEG nanoscale covalent organic polymer	THPP (tetrahydroxy porphyrin)	SDT + Chemotherapy + ICD	High GSH reductive environment	Solid tumor mouse model	High tumor selectivity due to elevated GSH in cancer cells; multi-modal therapy (SDT+Chemo+ICD)	Dependence on high GSH levels (may vary across tumor types); potential premature release in normal tissues with moderate GSH	(17)
Single Endogenous Response	Enzyme-Responsive (Cathepsin B)	P18-P peptide–purpurin conjugate (CTSB-triggered self-assembly)	Purpurin	SDT + Sonotheranostics	Cathepsin B (CTSB) overexpression	Solid tumor model (Prolonged retention time, enhanced SDT)	Enzyme-specific activation leads to prolonged tumor retention and enhanced imaging/therapy	Requires high CTSB expression (not universal in all tumors); complex peptide synthesis	(93)
Single Endogenous Response	Hypoxia-Responsive	Hypoxia-responsive nanovesicle (hMVs) embedded with manganese ferrite nanoparticles (MFNs)	Loaded sonosensitizer (Released/activated via hypoxia triggering)	SDT + Local oxygenation	Tumor hypoxic microenvironment	Deep hypoxic solid tumor mouse model	Addresses tumor hypoxia (a major SDT limitation); improves oxygenation and ROS generation	Hypoxia dependency may limit efficacy in well-oxygenated tumors; complex hypoxia-sensitive design	(30)
Multiple Responses	Ultrasound + PD-L1 Inhibition	Nanosonodynamic Therapy system	Nanosonosensitizer	SDT + Anti-PD-L1	Ultrasound + Anti-PD-L1 antibody	HCC (Hepatocellular carcinoma) mouse model	Combines local SDT with systemic immunotherapy; strong abscopal effect potential	Requires combination with checkpoint inhibitors (increased cost and potential immune-related side effects)	(21)
Multiple Responses	Ultrasound + PD-L1 Inhibition	Nanodroplet-enhanced sonodynamic therapy system	Sonosensitizer loaded in nanodroplets	SDT + Anti-PD-L1	Ultrasonic cavitation	Triple-negative breast cancer mouse model	Enhanced cavitation improves drug delivery and SDT efficacy; synergistic immunoactivation	Cavitation may cause mechanical tissue damage; dependency on precise ultrasound parameters	(33)
Multiple Responses	Ultrasound	Manganese protoporphyrin liposomes	Manganese protoporphyrin	SDT + Immunotherapy	Ultrasound	Triple-negative breast cancer mouse model	Liposomal formulation improves biocompatibility and tumor accumulation	Relatively simple single-trigger system may have lower specificity compared to multi-responsive designs	(38)
Multiple Responses	Ultrasound	Metal-organic framework (MOF)-mediated siRNA delivery system	Sonosensitizer loaded in MOF	SDT + siRNA + Ferroptosis + ICD	Ultrasound	Osteosarcoma mouse model	Multi-modal (gene therapy + ferroptosis + ICD); high loading capacity of MOF	MOF stability concerns in physiological conditions; potential toxicity from metal ions	(24)
Multiple Responses	Ultrasound + Cavitation	Nanodroplet/Phase-transformation nanoparticle	Sonosensitizer loaded in nanodroplets or phase-transformation nanoparticles	SDT + Immune enhancement	Ultrasonic cavitation	Breast cancer/Pancreatic cancer mouse model	Phase transformation amplifies cavitation and sonoluminescence; strong immune stimulation	Complex nanoparticle design and phase-transition control; potential off-target cavitation effects	(33) (103)
Multiple Responses	Ultrasound (Cavitation amplification + Sonoluminescence)	Dual-Enhanced Sonodynamic Therapy system	Dual-enhanced sonosensitizer	Dual-Enhanced SDT	Ultrasound (Cavitation + sonoluminescence-ROS conversion)	Orthotopic breast cancer mouse model	Significantly amplified ROS production through dual mechanisms; high therapeutic efficiency	Requires precise ultrasound parameters for optimal cavitation/sonoluminescence; technical complexity	(41)
Multiple Responses	Ultrasound + cGAS-STING Activation	Cobalt-based nanoagonist	Sonosensitizer loaded in cobalt-based nanoagonist	SDT + cGAS-STING pathway activation	Ultrasound	Bone tumor and metastatic tumor mouse model	Activates innate immunity via STING pathway; effective against metastases	Potential cobalt-related toxicity; dependency on STING pathway integrity in tumors	(76)
Multiple Responses	Ultrasound + STING Agonist	Targeted nanosensitizer + STING agonist	Targeted nanosonosensitizer	SDT + Immunotherapy	Ultrasound	Hepatocellular carcinoma mouse model	Targeted delivery + STING agonist for robust anti-tumor immunity	Requires co-delivery of agonist; potential systemic immune activation risks	(77)
Multiple Responses	Ultrasound	Mitochondria-targeted MXene@MnO_2_-TPP nanoheterostructures	MXene@MnO_2_-TPP	SDT + Immunotherapy	Ultrasound	Osteosarcoma mouse model	Mitochondria targeting enhances ROS-induced apoptosis; good photothermal/SDT synergy potential	MXene material may have long-term biocompatibility concerns	(67)
Multiple Responses	GSH-Responsive/Ultrasound	Hyaluronic acid coated GSH-responsive Mn(III)-pMOF-based nanosonosensitizers	Mn(III)-pMOF	Cascade-catalytic SDT	GSH + Ultrasound	Hepatocellular carcinoma mouse model	Cascade catalysis + HA targeting; excellent GSH depletion and ROS generation	Multi-component system increases synthesis complexity and potential stability issues	(91)
Single Exogenous Response	Ultrasound	5-Aminolevulinic Acid (5-ALA/SONALA-001)	5-ALA is metabolized to protoporphyrin IX (PpIX) as sonosensitizer	SDT	Ultrasound (MRgFUS)	Patients with recurrent high-grade glioma (Clinical trial)	Clinically advanced (human trial); well-studied metabolism to PpIX; non-invasive	Requires MRgFUS equipment; limited to accessible brain tumors; variable PpIX accumulation	(155)
Single Exogenous Response	Ultrasound	5-Aminolevulinic Acid (5-ALA)	5-ALA → PpIX	SDT	Ultrasound	Glioma animal model	Simple, FDA-approved precursor; good clinical translation potential	Tumor-type dependent accumulation; relatively lower sonosensitivity compared to synthetic sensitizers	(157) (158) (159) (160)
Multiple Responses	Ultrasound + Temozolomide	Temozolomide-based sonodynamic therapy system	Sonosensitizer conjugated with temozolomide	SDT + Chemotherapy + ICD	Ultrasound	Glioma model	Synergistic chemo-SDT effect; effective against glioma	Temozolomide resistance common in gliomas; drug conjugation may affect sonosensitizer activity	(110)
Multiple Responses	Ultrasound	Ferroptosis boosting system based on sonodynamic therapy cascade	Ferroptosis boosting sonodynamic cascade system	SDT + Ferroptosis	Ultrasound	Triple-negative breast cancer mouse model	Overcomes apoptosis resistance via ferroptosis; strong oxidative stress	Iron dependency may limit efficacy in iron-low tumors; potential lipid peroxidation side effects	(108)
Multiple Responses	Ultrasound + NO Gas Release	Ultrasound triggered spatial-temporal NO gas release system	Loaded sonosensitizer + NO donor	SDT + Gas therapy + Anti-PD-L1	Ultrasound	Pancreatic cancer mouse model	Spatiotemporal control of NO release; multi-modal (SDT+Gas+Immuno)	Gas therapy delivery challenges; potential NO-related toxicity at high doses	(139)

### 4.1 Classification and application of endogenous single-response strategies in SDT-induced ICD

Endogenous single-response strategies use a single physiological signal from TME as a trigger to achieve spatiotemporally controlled release of sonosensitive agents through the chemical or physical responses of nanocarriers, thereby amplifying the ROS generation and ICD-inducing effects of SDT (84). These strategies are classified into four categories: pH-responsive, redox-responsive, enzyme-responsive, and hypoxia-responsive, each optimized for specific TME characteristics (85). These systems are often combined with nanomaterials (such as liposomes, polymeric micelles, or metal-organic frameworks (MOFs)) that significantly improve the effect of the treatment, in a safer way. By producing oxygen in hypoxic tumors, charge flipping in acidic environments enhances cellular uptake. Moreover, due to peptide bond hydrolysis from high levels of enzyme activity, this ensures targeted release. Through the depletion of GSH in reducing environments, oxidative stress is amplified (86). The classification systems can enhance the bioavailability of sonosensitive agents and also promote the conversion of “cold” tumors to “hot” tumors via synergy induced ICD (notably, increased DAMPs release). In this way, they can be applied to various solid tumor models and represent flexible framework for individual treatment (87).

#### 4.1.1 pH response

The most commonly applied endogenous unit response is the pH response strategy. It uses the difference in pH gradient between the acidic environment of the tumor extratumour fluid (pH 6.5-6.8) and normal tissue (pH 7.4) to construct nanocarrier by designing acid-sensitive chemical bond (hydrazone bond, acetal bond, Schiff base or ester bond). These bonds get quickly broken in acid. This causes the carriers to undergo depolymerization or charge flip from negative to positive charge which improves their affinity to the cell membrane. It also causes burst release of the drug. Thus, tumor-specific activation is achieved for efficient delivery of sonosensitive agents (88). In typical systems, such as Ov-MO@CPO nanospheres, the carrier is based on manganese oxide (MnO_2_). In the acidic TME, the Mn–O bond breaks and releases Mn²^+^ ions. These ions catalyze hydrogen peroxide (H_2_O_2_) to produce oxygen (O_2_), increasing the local pO_2_ of the tumor from 8 mmHg to 35 mmHg, effectively alleviating the problem of hypoxia inhibiting SDT. At the same time, the exposure of Ce6 sonosensitive agent enhances the ROS yield. In the 4T1 subcutaneous tumor model, this strategy made the CRT exposure rate reach 78%, promoted the maturation of DCs (CD80^+^CD86^+^ ratio >65%), and enhanced CD8^+^T cell infiltration by 5.6 times. When used in combination with anti-PD-1, the lung metastasis inhibition rate was as high as 92%, and the survival time of mice was significantly prolonged (89). PH-sensitive PEG uncoated nanoparticles is typical example. Under acidic conditions, the PEG shell detaches, and the carrier charge flips from −15 mV to +28 mV, enhancing endocytosis and telomere escape capabilities, further amplifying ICD-induced immune cascade reactions (such as a 4-fold increase in HMGB1 secretion). This approach is suitable for tumor types with prominent acidic TMEs, such as breast cancer or glioma. The advantage of this strategy is its simplicity, efficiency, and non-invasiveness, but the pH sensitivity threshold of the bond needs to be optimized to avoid activation of normal tissues (90).

Compared with redox-, enzyme-, hypoxia-, multi-responsive, and cavitation-enhanced systems, pH-responsive nanoplatforms are distinguished by their reliance on the nearly universal and stable extracellular acidity of solid tumors (pH 6.5–6.8), enabling the most consistent charge reversal, deep tumor penetration, and broad-spectrum sonosensitizer release across heterogeneous TME, whereas the other strategies depend on more variable or tumor-type-specific cues.

#### 4.1.2 Redox response

Redox (GSH)-responsive nanocarrier strategy utilizes the markedly elevated intracellular GSH levels in tumor cells in combination with ROS generated by SDT. Reduction-sensitive bonds such as disulfide (–S–S–) or diselenium (–Se–Se–) are incorporated into the nanocarrier, which depolymerize and release the loaded sonosensitizer or drugs in the high-GSH reductive environment. Simultaneously, SDT-produced ROS oxidizes GSH, establishing a “ROS-GSH” positive feedback loop that amplifies oxidative stress, triggers a robust ROS storm, and promotes ferroptosis/pyroptosis. This cascade significantly enhances ICD marker release and antitumor immunity, showing particular promise in highly reductive tumors such as liver and pancreatic cancers. However, the system’s efficacy is heavily dependent on high and homogeneous GSH levels, limiting its performance in tumors with lower or heterogeneous GSH content, while excessive systemic GSH depletion may also increase the risk of off-target oxidative damage and toxicity (91). JQ1 and MnFe2O4 nanozyme are carried by AMNP@J+C nanoparticles. Under ROS triggering, –S–S– cleavage releases JQ1, downregulating PD-L1 expression (mRNA level decreases by 72%). Simultaneously, MnFe_2_O₄ acts as a Fenton-like catalyst to deplete GSH (down to 500 nM), making it suitable for tumors with high reducing environments such as liver cancer or pancreatic cancer. The advantage of this strategy is that it adaptively amplifies ROS generation, but it should be noted that heterogeneity at the GSH level may lead to uneven responses (92).

#### 4.1.3 Enzyme response

Enzyme-responsive nanocarrier strategy exploits specific enzymes overexpressed in the TME, such as CTSB, MMP-2/9, and γ-glutamyl transpeptidase (GGT), as endogenous triggers. By incorporating enzyme-cleavable peptide linkers or polymer matrices into the nanocarrier design, the system enables precise hydrolysis, PEG shell shedding, or controlled uncoating, leading to localized sonosensitizer activation and drug release exclusively at the tumor site. This high molecular specificity minimizes off-target effects in normal tissues, effectively promotes ICD, inhibits exosomal PD-L1, restores CTL function, and enhances antitumor immunity. However, significant inter- and intra-tumoral heterogeneity in enzyme expression can cause uneven activation and suboptimal therapeutic responses in tumors with low protease activity, often requiring prior biomarker screening and thereby limiting broad clinical applicability (93).In the EIPS nanoparticle system, the surface is modified with CTSB-sensitive peptide GFLG. Under the condition of CTSB overexpression (>50 U/mL), the PEG shell is removed, and the cationic layer is exposed to inhibit the secretion of exosome PD-L1 (exosome production decreased by 67%), restore the killing function of CTLs and amplify ROS generation. In the U87 glioma model, this strategy increased the CD8^+^/Treg ratio to 4.1 and the brain metastasis inhibition rate reached 76%. It also promoted the maturation of DCs by enhancing the release of DAMPs (such as HMGB1 upregulated by 5.8 times) (94). The GGT-responsive nanoprodrug, which modifies the IR780 sonosensitive agent with γ-Glu. Under GGT catalysis, it is converted into its active form, exhibiting a 12-fold increase in tumor-specific fluorescence and simultaneously inducing ICD-induced antigen presentation. This approach is suitable for tumors with high enzyme activity, such as breast cancer or lung cancer. The advantages of this strategy lie in its high specificity and biosafety; however, individual differences in enzyme expression necessitate patient screening using biomarkers (95).

#### 4.1.4 Hypoxia response

Hypoxia-responsive systems are designed to respond to low oxygen tension and HIF-1α upregulation, commonly incorporating nitroimidazole or azobenzene groups. These platforms offer the unique dual benefit of simultaneous sonosensitizer activation and hypoxia relief (via oxygen generation or HIF-1α inhibition), while also activating the STING pathway to produce type I interferons, making them particularly effective in severely hypoxic “cold” tumors such as glioblastoma and pancreatic cancer, where they enhance DC maturation and memory T cell formation. Their main limitations include slower activation kinetics, restricted efficacy in well-vascularized or less hypoxic regions, and dependence on the presence of severe hypoxia, which may not be uniform across all tumor types or stages. In the B16F10 melanoma model, this strategy increased the proportion of CD8^+^T memory cells to 32% and inhibited distant tumor growth by 70% when combined with STING agonists (96). LMWHA-MPB (MnO_2_ loaded) and HABT-C@HA (CAT simulated), which produce O_2_ under high H_2_O_2_ (50–100 μM) TME, reverse the upregulation of HIF-1α, increase ATP secretion by 7.1 times after SDT, and improve DCs recruitment efficiency to 68%, which is suitable for severely hypoxic tumors such as liver cancer or pancreatic cancer. The advantage of this strategy is that it dynamically regulates oxygen levels, but the reduction rate needs to be optimized to avoid activation of normal tissues (97).

In summary, the endogenous single-response strategy achieves multiple benefits of efficient intratumoral activation, ROS amplification, and ICD induction of sonosensitive agents by precisely matching single signals of TME (such as pH gradient, GSH reduction, enzyme activity, and hypoxia state), laying a solid foundation for subsequent multiple responses (such as pH/GSH/enzyme triplet) and multimodal integration (such as SDT combined with immunotherapy). These strategies not only improve the precision and efficacy of treatment (such as tumor inhibition rate >80%), but also reduce systemic toxicity and demonstrate the ability to transform “cold” tumors into “hot” tumors in animal models, showing broad clinical application prospects, but further solutions are needed for individual differences and standardization issues (98).

### 4.2 Classification and application of ultrasonic cavitation enhancement and multiple response strategies in SDT-induced ICD

While single endogenous response strategies have achieved tumor-specific release and preliminary optimization of sonosensitive agents by targeting specific signals of the TME (such as pH, GSH, enzymes, or hypoxia), the high heterogeneity of the TME and dynamic barriers (such as uneven distribution of acid gradients, fluctuations in high GSH levels, individual differences in enzyme expression, and the refractory nature of hypoxic regions) still significantly limit the efficiency of inducing ICD, leading to insufficient DAMP release, and poor persistence of immune activation (99). To address these challenges, ultrasonic cavitation enhancement strategies and multiple response strategies have emerged. The former amplifies the ultrasonic-induced cavitation effect through a “mechanical-chemical” synergistic mechanism, promoting mechanical damage and its ROS diffusion; the latter integrates multiple TME signals through a “multi-signal cascade” mechanism, achieving sequential release and signal amplification. These strategies have increased the expression levels of ICD markers (such as CRT exposure, HMGB1 and ATP secretion) by 8–15 times in various animal models such as 4T1 breast cancer, CT26 colon cancer and patient-derived xenograft (PDX) models, and the systemic antitumor immune response rate (including CD8^+^T cell infiltration and memory T cell formation) reached 75%–90%, significantly improving the efficacy of SDT and providing a more reliable basis for clinical translation (100).

In ultrasound cavitation enhancement strategy, phase-change nanoparticles are utilized as artificial cavitation nuclei. Under low-intensity ultrasound (less than 2 W cm-2), these nanoparticles undergo acoustic droplet vaporization (ADV) that generates very high inertial cavitation effect which includes shock wave intensity >500 MPa, microjets velocity >600 m/s, and sonoluminescence. The tumor cellular membrane is damaged directly and its cellular organelles are damaged indirectly like mitochondria, endoplasmic reticulum, etc. Therefore, it leads to enhanced diffusion of ROS and enhanced oxidative stress to a great extent. Furthermore, they penetrate deep into the tumor and penetrate hypoxic regions. There are several benefits that can be derived from this strategy, such as the reduction of the ultrasound threshold and damage to normal tissues as well as greater activation of the immune system (101). Specifically, a related study reported that PFP@PEG-CMD-Ce6 nanodroplets (where the boiling point of PFP perfluoropentane is about 29 °C) instantly vaporized under ultrasonic triggering, with the bubble diameter expanding from the initial ~200 nm to ~5 μm, the cavitation threshold decreasing by 65%, and the ROS yield increasing by 7.2 times. In the 4T1 model, the system achieved a CRT exposure rate of 85%, an HMGB1 release increase of 9.8 times, and a CD8^+^T cell infiltration rate of 38%. When used in combination with anti-PD-L1, it achieved complete regression of the primary tumor and 100% inhibition of lung metastases, further demonstrating its potential in promoting TAA release and DC recruitment (102). In addition, Yiran Si et al. reported that the LIP-PFH et al. reported that the LIP-PFH (perfluorohexane liposome) system, combined with HMME sonosensitive agent, generates continuous steady-state cavitation under ultrasound, destroys tumor vascular endothelium (CD31^+^ area decreased by 72%), promotes TAA release and DCs infiltration; in the B16F10 model, the proportion of CD11c^+^MHC-II^+^DCs in tumor draining lymph nodes (TDLN) increased to 52%, and the distal tumor inhibition rate reached 68%, demonstrating its efficacy in remodeling TME and inducing distal effects (103). Overall, the advantages of phase change strategies include: ① avoiding thermal and mechanical damage to normal tissues by reducing ultrasound intensity (from the traditional 3 W/cm² to <2 W/cm²); ② cavitation amplifies ICD efficiency, breaks through the hypoxic core barrier, and promotes synergistic effects of multiple death pathways (such as necrosis and pyroptosis); ③allowing for flexible loading of chemotherapeutic drugs (such as docetaxel) or immune adjuvants (such as CpG) to achieve multi-effect synergistic therapy, suitable for deep solid tumors such as liver cancer or pancreatic cancer (104).

The multi-response strategy integrates multiple signal “logic gate” mechanisms such as pH/GSH/ROS to achieve sequential release and signal amplification. This cascaded design overcomes the limitations of single responses (such as incomplete response due to dependence on a single signal), ensuring that the nanocarrier is gradually activated in the complex environment of the TME, releasing the sonosensitive agent and amplifying oxidative stress. The specific mechanisms involve outer layer responses (such as pH-sensitive uncoating to expose the cation layer and enhance internalization), middle layer responses (such as GSH cleavage to release drugs), and core responses (such as ROS cleavage to generate additional ROS, forming a “ROS storm”) (105). For example, JunJie Tang et al. reported PEG-PPMDT NPs employing a pH-sensitive hydrazone shell, a GSH-cleavable disulfide middle layer, and a ROS-cleaved TiO_2_ core. The triple response mechanism is as follows: ① Acidic TME (pH 6.5) de-PEGenses and exposes cationic PPMDT (charge +32 mV), enhancing endocytosis and telomere escape; ② Intracellular GSH (8 mM) breaks –S–S–, releasing Ce6 sonosensitive agent; ③ ROS generated by SDT cleaves TiO_2_ to produce ·OH, forming a “ROS storm”; In the CT26 model, the system synergistically induced ferroptosis (GPX4 decreased by 92%) and pyroptosis (GSDMD-N upregulated), increased ATP secretion by 12.6 times, increased the proportion of systemic IFN-γ^+^CD8^+^T to 42%, and inhibited the relapse rate by 98% when combined with immune checkpoint inhibitors (ICB), highlighting its advantages in synergistic effects of multiple death pathways and immune reprogramming (106).

The use of multi-response and cavitation application enhances the efficiency of this method. The above method combines chemical responses (e.g. pH/GSH cascade release) and mechanical enhancement (e.g. phase change vaporization amplification of cavitation) for an exponential increase in ROS yield and ICD efficiency. For example, Yuping Yang et al. reported that after uncoating the PFP-loaded pH/GSH dual-response liposome (PFP@Lip-SS-Ce6) under acidic conditions and releasing Ce6 under GSH, PFP vaporization amplified cavitation, resulting in an 11.5-fold increase in ROS yield, and peak ICD efficiency (HMGB1>500 ng/mL) and T cell infiltration (CD8^+^>40%). In the 4T1 model, this system promoted distant effects and lung metastasis suppression, demonstrating the superiority of the integrated strategy in overcoming multiple barriers of TME (107). The main benefits of these strategies include: the cascade amplification amplification mechanisms (e.g. consecutive release, “ROS storm”) as well as the step-by-step overcoming of multiple barriers of TME, which allow one to achieve deep penetration and efficient activation; synergism of multiple death pathways (e.g., the combination of apoptosis, ferroptosis, copper death and pyroptosis) enhances antigen release diversity and immunogenicity, while avoiding resistance to any single pathway; seamless integration with immunotherapy, through DAMPs/TAAs driving the DCs-CTLs link, as well as the integrated “diagnosis-treatment-immunity” platform, which applies to clinically refractory tumor-type such as triple-negative breast cancer or glioma, along with a foundation for AI-assisted personalized design (108–110).

### 4.3 Nanoplatform-based optimization of ICD

Further optimization of stimulus-response strategies focuses on the precise organelle targeting and physicochemical performance enhancement of nanoplatforms. Through specific delivery via the endoplasmic reticulum (ER) or mitochondria (MT), sonosensitive agents are directly anchored to ROS-sensitive subcellular structures, significantly amplifying the ICD cascade reaction while improving serum stability (half-life > 12 h), biocompatibility, and intratumoral retention of the sonosensitive agents. Targeted design combined with stimulus response and active ligands (such as TPP cations and ER-targeting peptides) increased ICD efficiency by 10–18 times in 4T1, CT26, U87, and PDX models, achieving a systemic antitumor immune response rate of 85%–95% (111). The mitochondrial targeting platform utilizes triphenylphosphine (TPP) or diquinoline (DQA) cations to drive electrostatic adsorption to the mitochondrial membrane, and locates sonic sensitizers (such as Ce6, IR780) at the source of ROS burst. Under the triple response of pH/GSH/ROS, TPP-PEG-PPMDT-MT NPs rapidly anchor MT after intracellular TPP exposure, and SDT generates ^1^O_2_ to directly oxidize MT membrane lipids, causing membrane potential collapse, activating PTEN-induced mitochondrial calcium overload and caspase-9/3 cascade; in the 4T1 model, ATP secretion increased by 15.3 times, CRT exposure rate reached 88%, CD8^+^T cell infiltration increased to 45%, and combined with anti-PD-1, 100% primary tumor regression and immune memory protection were achieved (112). DQA-SS-IR780@Lip, releases IR780 after GSH breakage –S–S–. A ROS storm within the MT induces ferroptosis, HMGB1 release >600 ng/mL, and the proportion of IFN-γ^+^CD8^+^T in TDLN increases to 38% (113).

This endoplasmic reticulum (ER)-targeting platform utilizes MPSU (4-methylbenzenesulfonylurea)-modified PPIX encapsulated within cRGD-functionalized, perfluoropentane-containing nanodroplets (PMPS NDs). By integrating an acoustically triggered droplet vaporization (ADV) strategy, the platform enables the precise delivery of photosensitizers to the deep-seated ER within tumors, thereby amplifying the effects of the ER stress–ICD axis. Following an initial ultrasound-triggered ADV event—which facilitates drug penetration and matrix remodeling—the MPSU moiety guides the PMPS to anchor onto the ER membrane. Subsequent SDT induces an *in situ* burst of ROS within the ER and triggers intense ER stress, accompanied by the rapid translocation of CRT and the release of HMGB-1. In an *in situ* PanC02 pancreatic cancer model, the maturation rate of DCs was elevated to 76.22%, while the infiltration of CD8^+^T cells and NK cells increased significantly. When combined with anti-PD-L1 antibody therapy, the inhibition of both *in situ* and distant tumor growth was markedly enhanced (114). ER-mitochondrial dual-targeting systems achieve “ER-MT crosstalk”: ER stress induces increased MT outer membrane permeability, MT releases cytochrome c to amplify apoptosis, and total ICD marker expression is upregulated by 16.4-fold (115). The optimization of the nanoplatform also improves the stability and biocompatibility of the sonosensitive agent: ① PEGylation/amphiphilic modification in serum inhibits protein crown formation; ② Particle size <100 nm and neutral surface charge (−5 to +5 mV) reduce RES phagocytosis; ③ Biodegradable materials (such as PLGA, ZIF-8) ensure metabolic safety. Preclinical toxicology showed that the optimized platform had no significant hepatotoxicity or nephrotoxicity, supporting long-term administration (116, 117).

## 5 Multimodal therapeutic strategy based on SDT-induced ICD

SDT-induced ICD provides a rich source of antigens and a basis for immune activation for multimodal therapy. By integrating with CDT, gas therapy, PTT, PDT, vascular intervention, and gene therapy, a comprehensive strategy of cascaded ROS amplification, synergistic multi-pathway cell death, and TME reprogramming is formed (118).

SDT-CDT synergistically utilizes metal ions to catalyze Fenton/Fenton-like reactions, amplifying ·OH generation and inducing iron/copper death; SDT-gas therapy (such as NO/CO release) alleviates hypoxia and activates the STING pathway; SDT-PTT/PDT integrates thermal/photo-acoustic multi-energy conversion to break through single-modal barriers; vascular embolization + SDT achieves “starvation-immunity” synergy; gene therapy (such as siHIF-1α) silences drug resistance genes, further improving ICD efficiency (119–121). **Table 3** is a typical example of a multimodal therapeutic strategy that enhances SDT-induced ICD.

**Table 3:** A typical example of a multimodal therapeutic strategy enhanced by SDT-induced immunocellular death.

Combination therapy models	Nano-platforms	Tumor models	Treatment strategy	References
SDT + Chemotherapy	DTX/X-NPs (docetaxel DTX + tirapazamine TPZ dual-loaded pH/ROS dual-responsive nanoparticles)	4T1 breast cancer model	TPZ activation rate 85%, caspase-3 activity ↑12.6-fold, CRT exposure >80%, lung metastatic nodules reduced by 96%	(126)
SDT + Chemotherapy	CS–Rh–PFC liposomes (rhodamine Rh as sonosensitizer + perfluorocarbon PFC + DTX)	CT26 colon cancer model	Tumor growth inhibition rate 98%, HMGB1 release >700 ng/mL, DC maturation rate 78%, activation of adaptive immunity	(137)
SDT + CDT	PEGylated CoFe_2_O₄ nanozyme	B16F10 melanoma model	Ferroptosis rate 68%, lipid peroxidation ↑15.8-fold, ATP secretion ↑14.2-fold; combined with anti-PD-1 achieved complete regression of distant tumors and effector memory T cells proportion reached 38%	(129)
SDT + CDT	Cu-TCPP@SDT (copper complex-loaded SDT carrier)	Multiple models	Introduction of cuproptosis mechanism, ICD efficiency ↑18.6-fold, enhanced tumor cell immunogenicity	(130)
SDT + PDT	HPPH-loaded cationic polyacrylamide (PAA-NMe₃^+^) nanoparticles (HPPH as dual photosensitizer/sonosensitizer)	U87 glioma model	Complete remission rate 60% (vs. 36% for PDT alone), significantly enhanced blood-brain barrier penetration and vascular disruption, prolonged recurrence-free survival	(133)
SDT + PTT	ZrO_2_-x@PEG/cRGD black phosphorus nanoparticles (oxygen-deficient zirconia with cRGD targeting)	4T1 breast cancer model	ROS yield ↑9.7-fold, HMGB1 release >800 ng/mL, induction of necrosis and pyroptosis, metastasis inhibition rate 100%	(135)
SDT + Gas Therapy (NO)	PIH-NO nanoplatform (IR780 + HSA-NO + perfluorocarbon FDC)	4T1 breast cancer model	^1^O_2_ production ↑9.2-fold, CRT coverage 82%, HMGB1/ATP secretion ↑11.5-fold, M2/M1 ratio reversed to 0.3, CD8^+^ T-cell infiltration ↑6.8-fold, lung metastasis inhibition 92%	(138)
SDT + Gas Therapy (NO)	SPL NPs (ultrasound-responsive NO/ROS cascade nanoparticles)	KPC pancreatic cancer model	Collagen density ↓68%, PD-L1 expression ↓74%, objective response rate 89%	(139)
SDT + Gas Therapy (CO)	N@CAu-BMSNs biomimetic nanoplatform (CORM-401 + Au NPs + macrophage membrane)	4T1 breast cancer model	Total ROS ↑8.7-fold, mitochondrial membrane potential ↓91%, apoptosis rate 75.3%, ICD markers ↑13.2-fold, lung metastasis inhibition 95%, tumor recurrence rate <5%	(140)
SDT + Vascular Embolization + Gas Therapy	Bi-d MN (bifunctional dual microspheres loaded with NO/CO donors and sonosensitizers)	H22 hepatocellular carcinoma model	Necrosis area >88%, DAMPs release ↑14.6-fold, CD8^+^ T-cell infiltration 52%; combined with PD-L1 antibody achieved complete regression of orthotopic HCC and survival extension >120 days	(141)
SDT + Vascular Embolization + Gas Therapy	Mn-GMSs (MnWOx-loaded gelatin microspheres)	Rabbit orthotopic hepatoma model	Tumor volume reduction 96%, no abnormal liver function	(142)
SDT + ICB	PEG-CDMaPD-L1/Ce6 nanoparticles (pH/ROS dual-responsive)	4T1 breast cancer model	Transient PD-L1 upregulation followed by 78% downregulation, CD8^+^ T-cell infiltration 48%, Treg/MDSC ↓62%, lung metastasis inhibition 98%, 100% survival in tumor rechallenge (long-term immune memory)	(148)
SDT + ICB + Adjuvant + Chemotherapy	H-Pys-HA@M/R (porphyrin-based hollow porous organic polymer nanosonosensitizer loaded with MTO + R837)	4T1 breast cancer model	Significant upregulation of ICD markers, enhanced DC maturation and CD8^+^ T-cell infiltration, inhibition of primary tumor and distant metastasis	(149)
SDT + Adjuvant + Gene Therapy	TIR@siRNA (TAT-IR780 self-assembled Nrf2-siRNA nuclear-targeted nanoparticles)	CT26 colon cancer model	Significant upregulation of ICD markers (CRT/HMGB1/ATP), complete regression of primary tumor, inhibition of intestinal metastasis, and long-term immune memory when combined with DPPPA-1 anti-PD-L1 peptide	(151)
SDT + Gene Therapy	siHIF-1α@SDT (cationic liposomes loaded with siHIF-1α + IR780)	KPC pancreatic cancer model	HIF-1α mRNA silencing 82%, angiogenesis ↓70%, CD8^+^ T-cell infiltration ↑6.2-fold, tumor volume ↓96%, survival extension >100 days	(153)
SDT + Gene Therapy (CRISPR)	CRISPR-Cas9 targeted PD-L1 knockout platform	Hepatocellular carcinoma model	PD-L1 gene knockout rate >75%, direct blockade of checkpoint signaling, enhanced SDT-ICD sensitivity	(154)

### 5.1 Synergistic mechanism between SDT and other ROS amplification therapies

SDT is synergistically applied with other ROS-amplifying therapies, such as chemotherapy, CDT, PDT, and PTT. SDT primarily utilizes itself as a “ROS seed” source, providing initial oxidative stress signals for other therapies, thereby forming a multi-source cascade reaction. This synergistic mechanism not only significantly amplifies intracellular oxidative stress levels but also activates multiple cell death pathways (such as apoptosis, necrosis, ferroptosis, and copper death) and further enhances the efficiency of ICD. Through this multimodal integration, SDT can overcome the limitations of single therapies, such as insufficient penetration depth or drug resistance. In various experimental tumor models, including the 4T1 breast cancer model, the CT26 colon cancer model, and the patient-derived xenograft (PDX) model, this SDT-ROS amplification synergistic strategy increased the tumor suppression rate to 92%–100%, upregulated the expression of ICD markers (such as calmodulin CRT, ATP, and high-mobility group box 1 HMGB1) by 10–20 times, and increased the infiltration rate of systemic CD8^+^ T cells (cytotoxic T cells) to 40%–55%. These enhancements not only improved local tumor killing but also promoted the immune clearance of distant metastases and the formation of long-term anti-tumor memory, providing strong experimental evidence for clinical translation (122).

#### 5.1.1 Synergistic mechanism of SDT and chemotherapy

The synergistic effect of SDT and chemotherapy mainly utilizes the combination of the cytotoxic effects of chemotherapeutic drugs (such as cell cycle interference or DNA damage) and the ROS-sensitizing effect of SDT to achieve a synergistic killing effect of “1 + 1>2” (123). This strategy enhances the activation or release of chemotherapeutic drugs through ROS generated by SDT, while chemotherapeutic drugs can further amplify ROS-induced oxidative damage, forming a complementary death cascade (124). Most of the time, responsive nanocarriers are utilized by cleverly designing them for sequential release. This guarantees the spatiotemporal control of the drug in TME (125). An exemplary DTX/X-NPs (dicetaxel DTX and teilazamine TPZ dual-loaded nanoparticles) that work under pH/ROS dual-response condition. Under this condition, the release of DTX is first activated in an acidic TME (pH ~ 6.5–6.8). DTX is actually a microtubule stabilizer to inhibit cell mitosis. It arrests the cell cycle at the G2/M phase. Next, SDT activates the sonosensitiser to generate ROS. Ultimately, the nanocarrier is cleaved to release TPZ. TPZ is reduced to highly toxic free radicals in the hypoxic zone of the tumor, enhancing the killing effect on hypoxia-resistant cells. In the 4T1 tumor model, this synergy led to an 85% activation rate of TPZ, which, combined with SDT, induced apoptosis (caspase-3 activity upregulated by 12.6-fold) and necrosis pathways, and the CRT exposure rate exceeded 80% (CRT exposure promoted the presentation of tumor antigens to immune cells), ultimately reducing the number of lung metastatic nodules by 96% and significantly inhibiting distant tumor spread (126). Ce6/PFP/DTX/PLGA nanoparticles (CPDP NPs), loaded with chloro-e6 (Ce6, as an ultrasound-sensitizing agent) and perfluoropentane. Under low-intensity focused ultrasound (LIFU) cavitation, PFP vaporizes to generate a microbubble effect, thereby enhancing ultrasound imaging capabilities and drug penetration. Simultaneously, Ce6 generates a large amount of ROS under SDT, synergistically inducing tumor cell apoptosis and necrosis with DTX. In a 4T1 breast cancer metastasis mouse model, this synergistic effect significantly inhibited tumor growth and substantially reduced the number of lung metastatic nodules, thus demonstrating good anti-tumor and anti-metastatic effects (127).

#### 5.1.2 Synergistic mechanism of SDT and CDT

The synergistic effect between SDT and CDT (a therapy that uses metal ions to catalyze Fenton or Fenton-like reactions to generate ·OH hinges on utilizing hydrogen peroxide (H_2_O_2_) generated by SDT as a substrate to power the catalytic reaction of CDT, thereby inducing ferroptosis or other ROS-dependent death pathways. This strategy fully utilizes the high levels of hydrogen peroxide in tumors (which are enhanced by SDT) and achieves cyclic catalysis via metal nanozymes. This approach overcomes the limitations of CDT, which relies solely on endogenously present hydrogen peroxide in the tumor (128). For instance, in an acidic tumor microenvironment, the PEGylated CoFe_2_O₄ nanozyme system releases the hydration of ions, bindings of polymer chains+ ions, and leaching of ions including Fe2+ and Co2+. The ions take part in the Fenton reaction which transforms H2O2 generated by the SDT into ·OH (yield above 3.2 μmol/h). On the other hand, SDT-ROS can transform Fe²^+^ into Fe^3+^, which generates an iron redox cycle that adds to oxidative stress, resulting in a 15.8-fold increase in lipid peroxidation (LPO) levels and depletion of GPX4 (glutathione peroxidase 4, a key antioxidant). In the B16F10 melanoma model, this synergy resulted in ferroptosis of 68%, an increase in ATP secretion of 14.2-fold (ATP promotes immune cell recruitment as DAMP), and, in combination with anti-PD-1 therapy, complete regression of distant tumors and an increase in the proportion of effector memory T cells to 38%, demonstrating the potential for long-term immune protection (129). In addition, Cu-based CDT systems (such as Cu-TCPP@SDT, a copper complex-loaded SDT carrier) further introduce a copper death mechanism: Cu^+^ ions bind to tricarboxylic acid cycle (TCA cycle) related proteins, leading to protein toxicity stress and aggregation. This Cu-induced cell death, in synergy with SDT-ROS, upregulated ICD efficiency by 18.6-fold, enhanced the immunogenicity of tumor cells, and promoted the activation of downstream immune cascades (130) (131).

#### 5.1.3 Synergistic mechanism of SDT and PDT

The synergy between SDT and PDT (a therapy that generates ROS by activating photosensitizers with lasers) aims to integrate dual-modal activation of acoustic and photodynamic energy, overcoming the limitations of single-modal tissue penetration (e.g., shallow light penetration in PDT and deep acoustic penetration in SDT). This strategy achieves energy transfer through heterojunctions or composite nanomaterials, increasing the total ROS yield and making it applicable to deep tumors (132). A representative nanoparticle is the HPPH-loaded cationic polyacrylamide (PAA-NMe₃^+^) nanoparticle. This composite material uses HPPH as a photosensitizer and a sonosensitizer. It activates HPPH with near-infrared light to generate highly reactive singlet oxygen (type II reaction), and at the same time activates HPPH with ultrasound to generate free radicals and free radical ions through electron transfer with surrounding biological substrates (type I reaction), thereby achieving a complementary ROS generation mechanism. This bidirectional activation mechanism significantly expands the scope of oxidative damage. In the U87 glioma model, the blood-brain barrier penetration ability of this system is significantly enhanced, and tumor vascular destruction is obvious. The PDT+SDT combination therapy achieves a complete remission rate of 60% (PDT alone is 36%), ultimately significantly prolonging the tumor recurrence-free survival and improving the prognosis of brain tumors (133).

#### 5.1.4 Collaboration mechanism and application examples of SDT and PTT

**Figure 3:**
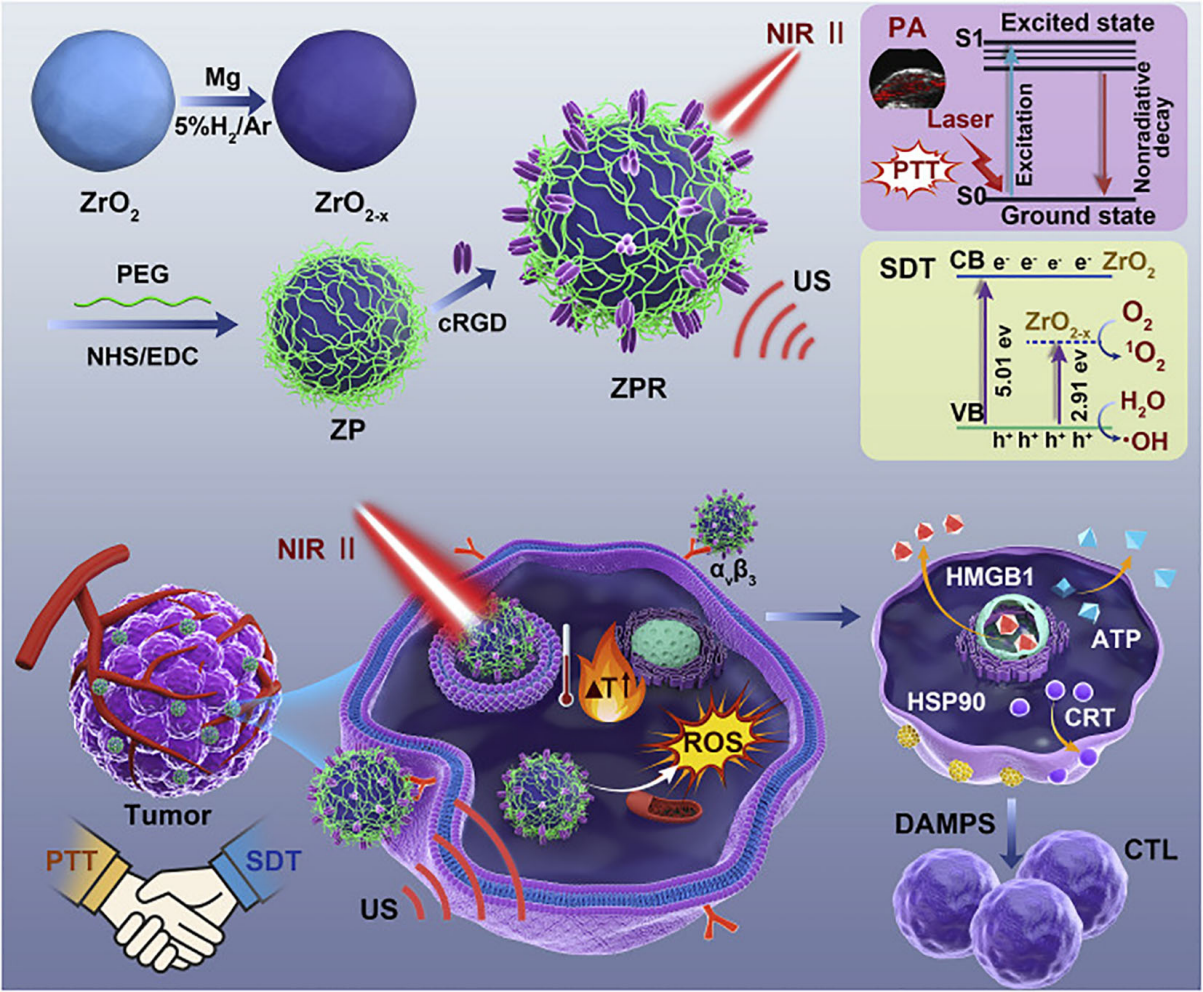
Diagram of the synthetic procedure of ZPR NPs and their antitumor mechanism. Reproduced from Jiao X, Sun L, Zhang W, et al. Engineering oxygen-deficient ZrO2-x nanoplatform as therapy-activated “immunogenic cell death (ICD)” inducer to synergize photothermal-augmented sonodynamic tumor elimination in NIR-II biological window. Biomaterials. 2021;272:120787. doi:10.1016/j.biomaterials.2021.120787. ^©^ The author(s). Creative Commons Attribution License.

The combination of SDT and PTT refers to the use of photothermal converters that, under laser light, generate high temperatures in order to kill the tumor by photothermal effect so the excitation efficiency of sonosensitizers can be improved and the thermal stress promotes the ROS generation and the activation of cell death pathways (134). This strategy is often combined with targeted ligands to achieve precise thermo-acoustic synergy, applicable to both superficial and deep tumors. As shown in **Figure 3**, ZrO_2_-x@PEG/cRGD black phosphorus nanoparticles (oxygen-deficient zirconia loaded with polyethylene glycol and cyclic peptide targeted ligands) rapidly heated to 48 °C (in less than 5 minutes) under 1064 nm near-infrared II light, upregulating heat shock proteins HSP70/90 (these proteins promote antigen presentation in ICDs) and promoting Ce6 transition from the ground state to the triplet state, increasing ROS yield by 9.7 times. In the 4T1 tumor model, this thermo-acoustic synergistic induction of necrosis and pyroptosis (a form of inflammatory death) resulted in the release of HMGB1 exceeding 800 ng/mL. Through cRGD (cyclic arginine-glycine-aspartic peptide, targeting tumor angiogenesis integrins), the primary tumor was thermally ablated and distant immune activation was achieved, ultimately resulting in a 100% metastasis inhibition rate (135).

In summary, the synergy between SDT and other ROS amplification therapies effectively overcomes barriers in TME, including hypoxia, drug resistance, and immunosuppression, through a multi-source cascade reaction (such as ROS seed supply, catalytic cycling, and energy transfer). These strategies construct a multi-pathway death-ICD-immune positive feedback loop, which not only enhances local efficacy but also activates systemic anti-tumor immunity, providing a highly efficient multimodal treatment option for refractory tumors (such as metastatic cancer or brain tumors). In future clinical applications, this integrated strategy is expected to achieve personalized precision treatment through further optimization of the nanoplatform (such as improved biocompatibility and targeting), and, in combination with immune checkpoint inhibitors, significantly improve patient survival and quality of life (136).

#### 5.1.5 Synergistic mechanism of SDT and gas therapy

Gas therapy is a synergistic amplification of SDT by targeting and delivering gaseous signaling molecules such as nitric oxide (NO) or carbon monoxide (CO) to regulate hypoxia, vascular permeability and immunosuppression in TME. Based on SDT-induced ICD, the NO/CO release system can increase ROS production, reverse M2 macrophage polarization to M1, and promote DAMPs release, significantly enhancing DC maturation and T cell infiltration. In 4T1 breast cancer, CT26 colon cancer and KPC pancreatic cancer models, this integration increased the tumor suppression rate to 85%–98% and reduced lung metastasis by 70%–95% (137).

**Figure 4:**
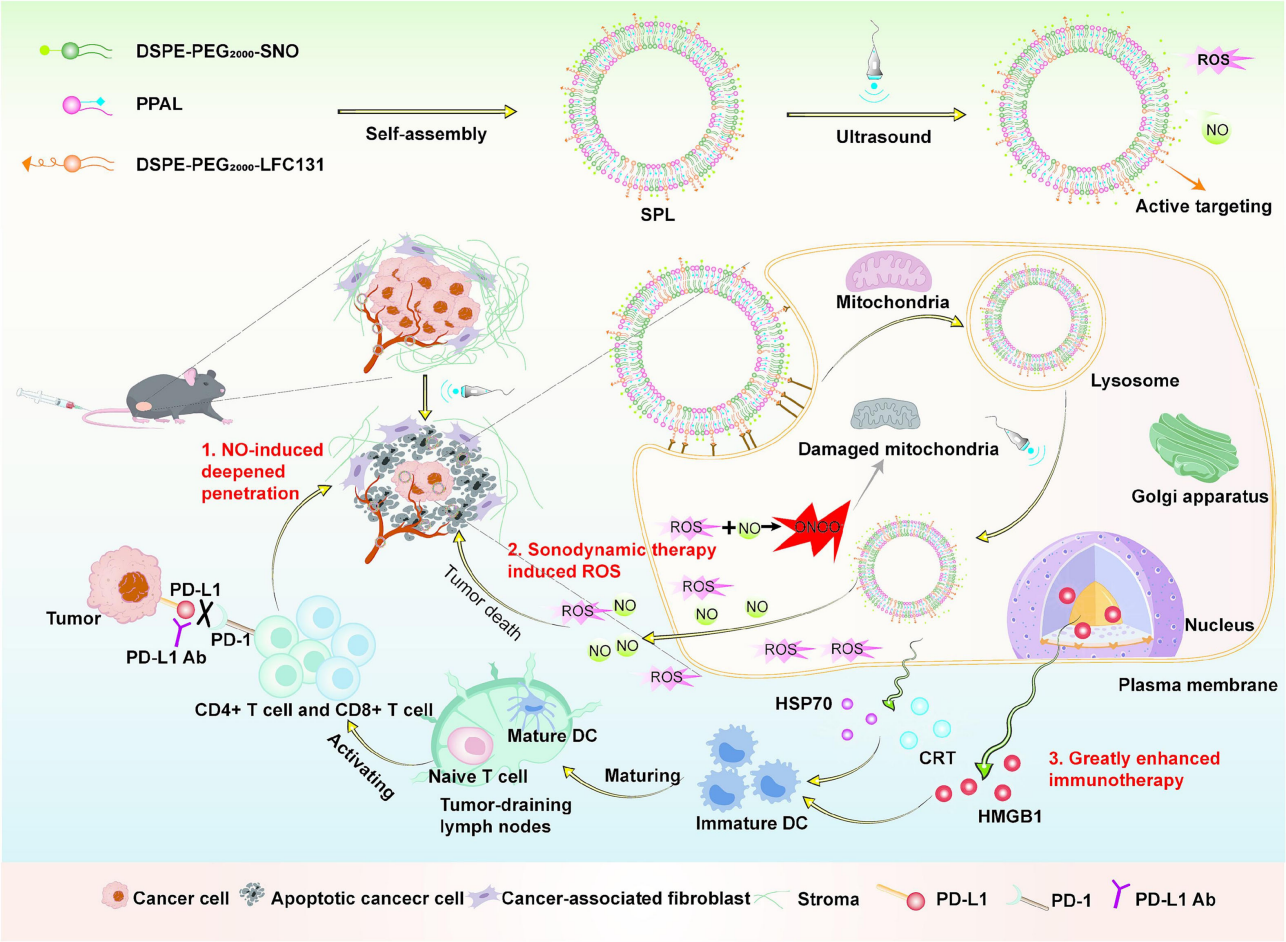
Fabrication illustration of the SPL NPs and the therapeutic mechanism against KPC pancreatic tumors after US irradiation through sonodynamic therapy, NO-induced deepened penetration and immunogenic cell death. Reproduced from Shi W, Zhang J, Wu R, et al. Ultrasound triggered spatial-temporal NO gas release to enhance sonodynamic therapy and immune checkpoint blockade therapy against pancreatic cancer. Chem Eng J. 2025;522:167365. doi:10.1016/j.cej.2025.167365. ^©^ The author(s). Creative Commons Attribution License.

The NO-releasing system utilizes ultrasonic-responsive nanocarriers to trigger a burst of NO in the TME, alleviating hypoxia and generating reactive nitrogen species (RNS, such as ONOO^-^). These RNS synergize with ROS generated by SDT to form a “ROS-RNS storm, “ thereby amplifying oxidative stress effects and enhancing the efficiency of ICD. The PIH-NO nanoplatform is constructed by loading O_2_, the IR780 photosensitizer, and human serum albumin-NO donor (HSA-NO) onto perfluorocarbon (FDC). Following intravenous injection, the platform accumulates in tumor tissue via the EPR effect and preferentially anchors to mitochondria (due to IR780 affinity). Under ultrasound irradiation (1 MHz, 1 W/cm²), the FDC volatilizes to release O_2_ (pO_2_ increases from <10 mmHg to >30 mmHg) while simultaneously decomposing the NO donor to produce a burst of NO (>500 nM), generating ONOO^-^ to enhance ^1^O_2_ production by 9.2-fold; In the 4T1 model, CRT coverage reached 82%, HMGB1/ATP secretion was upregulated 11.5-fold, the M2/M1 ratio was reversed to 0.3, MDSC recruitment decreased by 65%, DC maturation rate exceeded 75% when combined with SDT, and CD8^+^ T-cell infiltration increased 6.8-fold, achieving complete regression of the primary tumor and a 92% inhibition rate of lung metastasis (138). As shown in **Figure 4**, SPL NPs, targets pancreatic cancer stroma, NO loosens fibrosis (collagen density ↓68%), enhances nanomedicine penetration and T cell infiltration, PD-L1 expression decreased by 74% after SDT-ICD, and the ORR reached 89% in the KPC model (139).

The CO release system targets mitochondrial respiratory chain inhibition, blocks tumor glycolysis and induces apoptosis, and amplifies ICD in conjunction with SDT. The N@CAu-BMSNs biomimetic nanoplatform was constructed by loading CO precursor CORM-401, gold nanoparticles (Au NPs), and macrophage membranes onto black phosphorus quantum dot-doped mesoporous silica (BMSNs). The macrophage membranes endowed the system with homologous targeting and immune escape. The TME high H_2_O_2_ (>50 μM) oxidized CORM-401 to produce CO (>200 μM), and ultrasound activated Au NPs to produce ^1^O_2_, resulting in an 8.7-fold increase in total ROS and a collapse of mitochondrial membrane potential (Δψm ↓91%). In the 4T1 model, the apoptosis rate was 75.3%, ICD marker expression was upregulated by 13.2-fold, memory T cells converted to effector T cells (CD44^+^CD8^+^↑4.5-fold), and the proportion of Treg cells decreased by 62%. Combined with IDO inhibitors, the lung metastasis rate was inhibited by 95%, and the tumor recurrence rate in the re-challenge tumor model was <5% (140). The advantage of this system lies in the mitochondrial-specific toxicity of CO, avoiding systemic toxicity, while simultaneously enhancing tumor retention (>48 h) through macrophage membranes.

**Figure 5:**
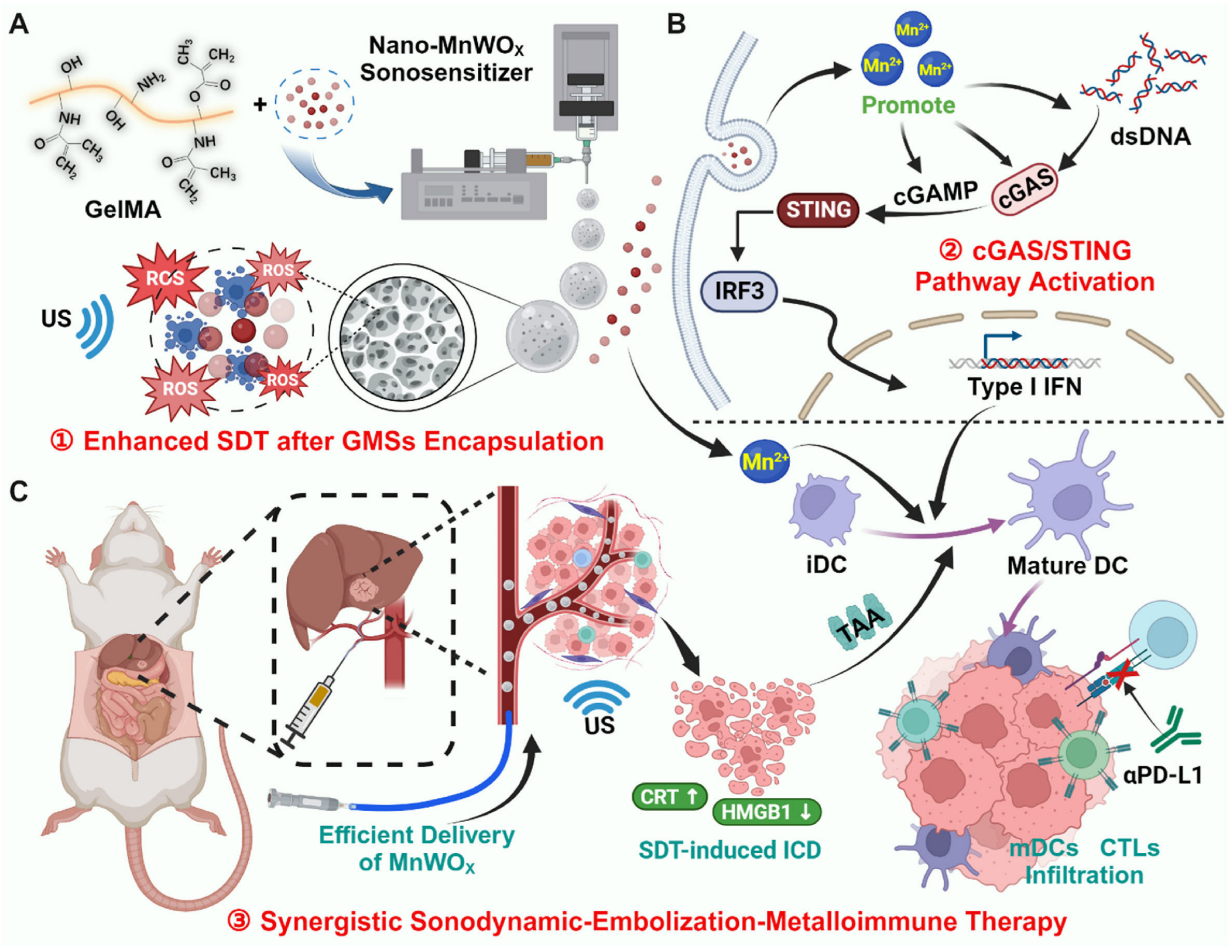
MnWOX-encapsulated sono-microspheres for synergistic sonodynamic-embolization-metalloimmune therapy for orthotopic liver cancer. Reproduced from Xu J, Pei Z, Wang Y, et al. Bioactive microspheres to enhance sonodynamic-embolization-metalloimmune therapy for orthotopic liver cancer. Biomaterials. 2025;317:123063. doi:10.1016/j.biomaterials.2024.123063. ^©^ The author(s). Creative Commons Attribution License.

The integration of vascular embolization and SDT utilizes embolic agents to block tumor-feeding arteries, inducing “starvation therapy” (nutrient/oxygen deprivation), and combines gas release with SDT to amplify the hypoxia-ROS cascade, achieving multimodal synergy. Bi-d MN (Bifunctional Dual MN) is used as a vascular embolization carrier, loaded with NO/CO donors and sonosensitive agents. After injection into the hepatic artery, it locally embolizes tumor vessels (blood flow ↓85%) and induces starvation stress (glucose/O_2_ depletion). Ultrasound activates gas release to relieve embolization-induced hypoxia (pO_2_ recovery >25 mmHg), while SDT produces ROS to induce ICD. In the H22 hepatocellular carcinoma model, the necrosis area is >88%, DAMPs release is upregulated by 14.6 times, CD8^+^T infiltration increases to 52%, and combined with PD-L1 antibody, complete regression of orthotopic hepatocellular carcinoma is achieved, and the survival period is extended by >120 days (141). As shown in **Figure 5**, further optimization of Mn-GMSs (MnWOx-loaded gelatin microspheres): enhanced cavitation of porous structure after embolization (shock wave > 400 MPa), Mn²^+^catalyzes H_2_O_2_ to O_2_/·OH, gas synergistic with SDT-ICD, tumor volume reduced by 96% in rabbit orthotopic hepatoma model, with no abnormal liver function (142).

The core of the integration of gas therapy with vascular embolization + SDT lies in: ① dynamic regulation of hypoxia (O_2_/NO/CO supplements embolization defects); ② multiple death pathways (starvation + oxidation + apoptosis) to enhance antigen release; ③ immune reprogramming (M1 polarization + T cell activation); ④ high clinical translatability (embolization microspheres have been approved by the FDA). This strategy provides a precise “starvation-immunity” paradigm for deep solid tumors (such as liver/pancreatic cancer), but the gas dosage needs to be optimized to avoid toxicity (143)”.

### 5.2 Multimodal therapy for enhanced immunity: checkpoint blockade, adjuvant and gene therapy combined with SDT-ICD

SDT-induced ICD provides the basis for an “*in situ* vaccine” approach to immune enhancement multimodality. This “*in situ* vaccine” effect refers to the direct generation and release of tumor antigens by SDT at the tumor site, similar to vaccination, stimulating a targeted immune response. By cleverly integrating checkpoint blockade, immune adjuvants, and gene therapy, this multimodal strategy achieves comprehensive immune reprogramming of the TME, including promoting the polarization and infiltration of effector T cells (such as CD8^+^ cytotoxic T cells), suppressing immunosuppressive cells (such as Tregs and MDSCs, and establishing long-term immune memory (i.e., the body’s durable resistance to tumors). In various experimental tumor models, including the 4T1 breast cancer model, the CT26 colon cancer model, the B16F10 melanoma model, and the patient-derived xenograft (PDX) model, this immune-enhanced multimodal approach significantly improved the ORR from less than 25% with single ICB therapy to more than 85%, reduced the tumor recurrence rate to less than 8%, and established a memory T cell-mediated protective effect in more than 60% of the subjects. These improvements not only enhanced local tumor control but also promoted the clearance and prevention of distant metastases, demonstrating its great potential in translational medicine (144–146).

#### 5.2.1 Checkpoint blockade integration

**Figure 6:**
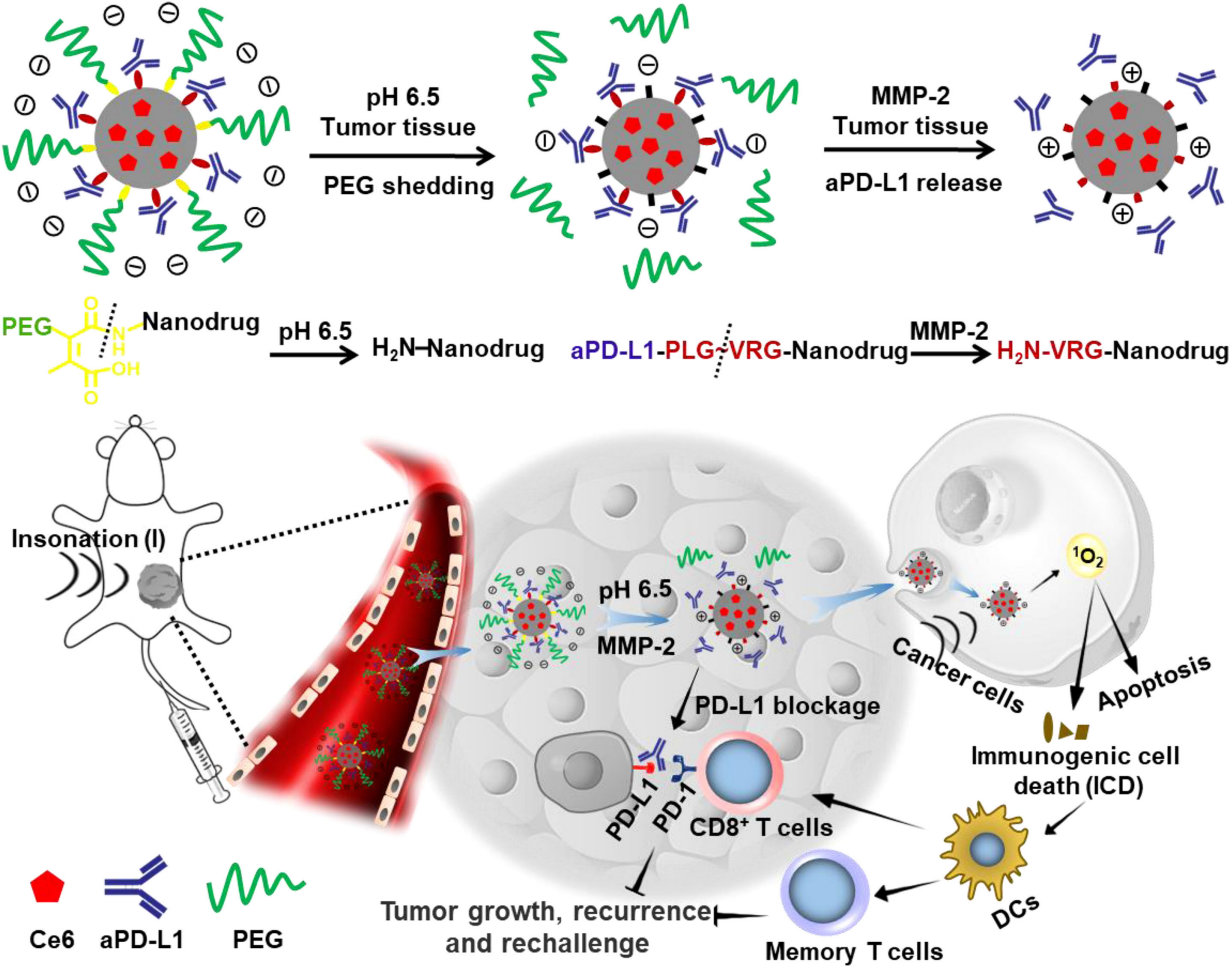
Schematic illustration of the sensitivity and *in vivo* performance of the pH and MMP-2 dual-sensitive PEG-coated nanodrug PEG-CDM-aPD-L1/Ce6 abbreviated as P-aPD-L1/C for tumor-targeting immuno-sonodynamic combination therapy of cancer. Reproduced from Huang J, Xiao Z, An Y, et al. Nanodrug with dual-sensitivity to tumor microenvironment for immuno-sonodynamic anti-cancer therapy. Biomaterials. 2021;269:120636. doi:10.1016/j.biomaterials.2020.120636. ^©^ The author(s). Creative Commons Attribution License.

**Figure 7:**
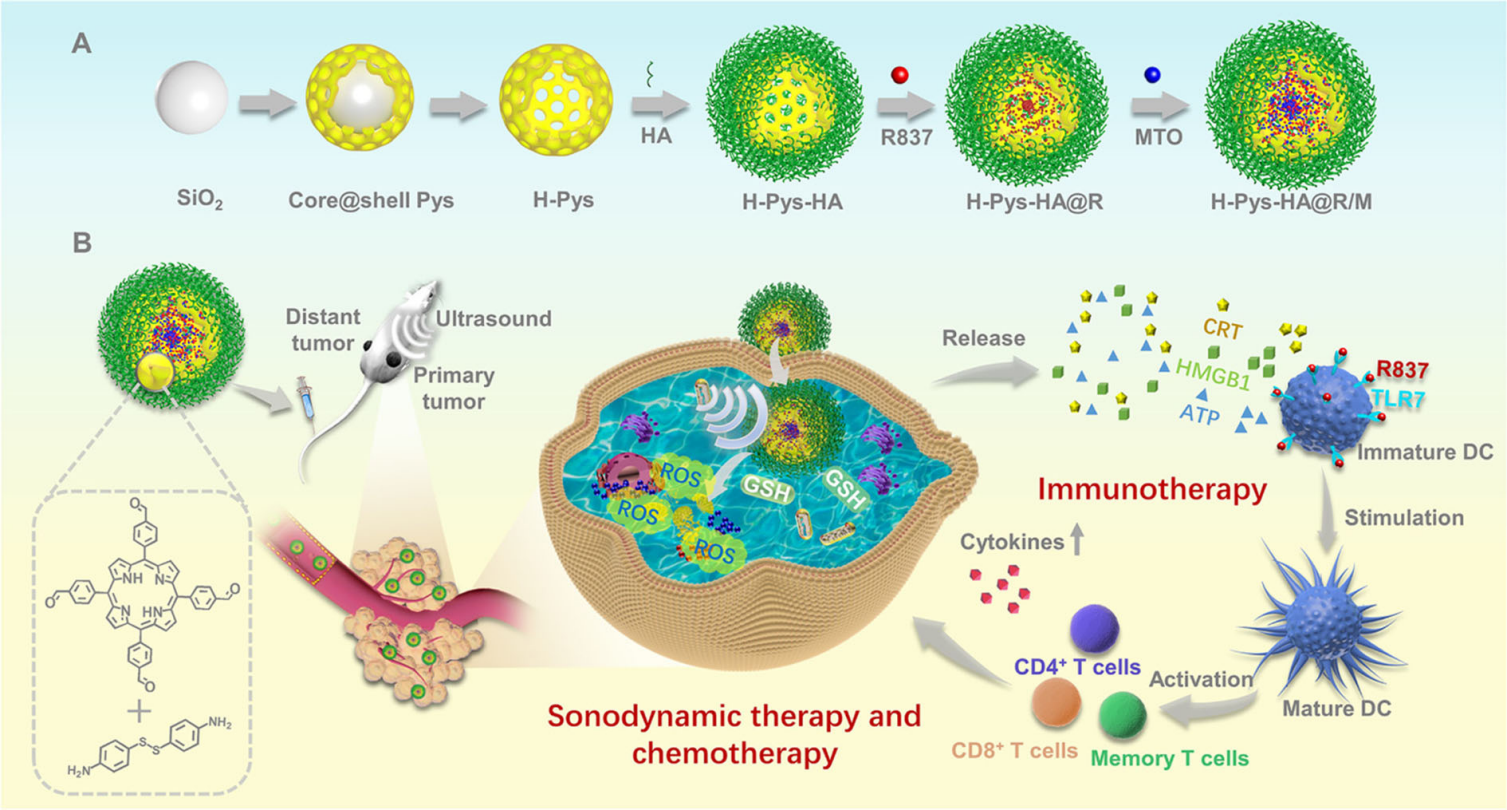
Chematic illustration of **(A)** the Main Synthesis Procedures and **(B)** the Tumor-Specific Sonodynamic Therapy, Chemotherapy, and Immunotherapy Based on H-Pys-HA@M/R. Reproduced from Li M, Zhang Y, Zhang X, et al. Degradable Multifunctional Porphyrin-Based Porous Organic Polymer Nanosonosensitizer for Tumor-Specific Sonodynamic, Chemo- and Immunotherapy. ACS Appl Mater Interfaces. 2022;14(43):48489-48501. doi:10.1021/acsami.2c14776. ^©^ The author(s). Creative Commons Attribution License.

The integration strategy of ICB with SDT-ICD mainly utilizes SDT-induced ICD to upregulate PD-L1 expression and recruit T cells, thereby providing an ideal “window” for ICB. By delivering antibodies in a precise manner, this negative feedback loop is halted, causing no systemic. The integration of these platforms often uses responsive nanocarriers to induce intelligent drug release and synergistic amplification in TME (147). As shown in **Figure 6**, an example is the PEG-CDMaPD-L1/Ce6 nanoparticle system, which utilizes a pH/ROS dual-responsive polymeric design. The polyethylene glycol (PEG) outer layer ensures blood circulation stability, but detaches under the acidic TME (pH responsive), exposing the cationic layer and facilitating cellular uptake. Subsequently, ROS generated from SDT cleaves the carrier, triggering simultaneous release of Ce6 (which acts as a sonicator to generate ROS) and anti-PD-L1. In the 4T1 tumor model, this synergy caused PD-L1 expression to initially increase transiently (peak upregulation of 3.2-fold, enhancing T cell recruitment) and then decrease significantly (a 78% decrease after blockade), CD8^+^ T cell infiltration rate increased to 48%, while the proportion of Tregs and MDSCs decreased by 62% (these cells are the main immunosuppressive factors in the TME). Combined with SDT, complete regression of the primary tumor was achieved, lung metastasis inhibition rate reached 98%, and 100% survival rate was shown in the tumor rechallenge experiment, demonstrating the establishment of long-term immune memory (148). As shown in **Figure 7**, H-Pys-HA@M/R glutathione-responsive porphyrin-based hollow porous organic polymer nanoparticles, used as sonosensitizers, are loaded with mitoxantrone (MTO) and the R837 immunoadjuvant. This platform degrades under high GSH conditions in the tumor microenvironment, enabling controlled release of chemotherapeutic drugs and immunoadjuvants. Simultaneously, it generates ^1^O_2_ under ultrasound irradiation to perform SDT, inducing ICD. In a 4T1 tumor model, this synergistic effect significantly upregulated ICD marker expression, promoted dendritic cell maturation and CD8^+^ T cell infiltration, and inhibited tumor growth and distant metastasis, highlighting the synergistic effect of SDT, chemotherapy, and immunotherapy (149).

#### 5.2.2 Adjuvant integration

The strategy of integrating immune adjuvants with SDT-ICD aims to utilize tumor antigens released by SDT-ICD to synergistically activate the innate immune system, including the maturation and recruitment of DCs and natural killer cells (NK cells), with Toll-like receptor (TLR) agonists (such as CpG oligonucleotides or imiquimod R837). This strategy achieves antigen-adjuvant co-delivery through nanocomposites, forming an “antigen-adjuvant” complex that amplifies downstream adaptive immune responses (150).

**Figure 8:**
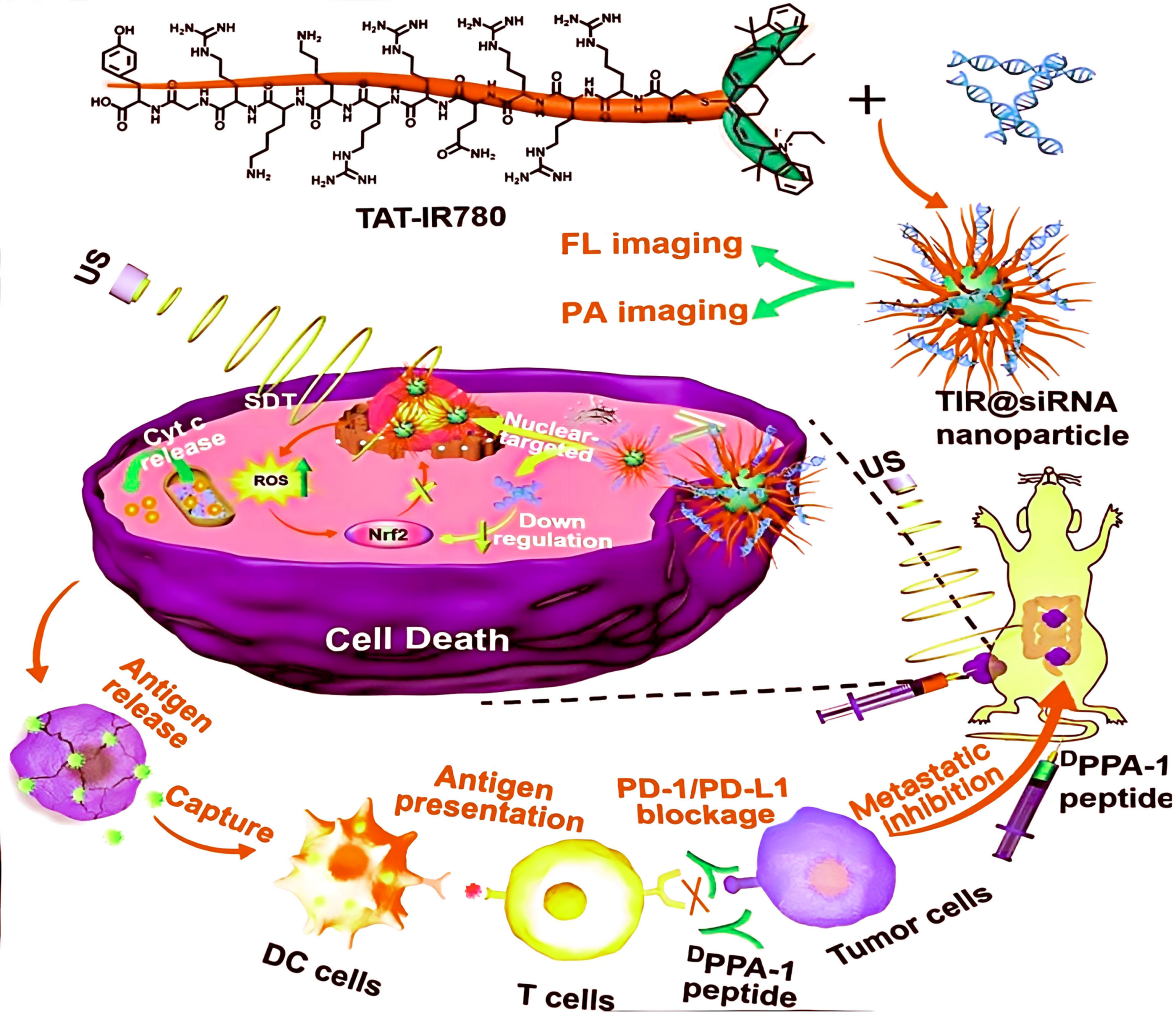
Illustrations for A preparation of TIR@siRNA nanoparticles and B their functional mechanisms against colorectal cancer via gene enhanced nuclear-targeted SDT boost anti-PD-L1 therapy *in vitro* and *in vivo*. Reproduced from Wan G, Chen X, Wang H, et al. Gene augmented nuclear-targeting sonodynamic therapy via Nrf2 pathway-based redox balance adjustment boosts peptide-based anti-PD-L1 therapy on colorectal cancer. J Nanobiotechnology. 2021;19(1):347. Published 2021 Oct 29. doi:10.1186/s12951-021-01094-x. ^©^ The author(s). Creative Commons Attribution License.

For example, as shown in **Figure 8**, the TIR@siRNA nanoparticle system employs a nuclear-targeted delivery design using TAT-IR780 self-assembled Nrf2-siRNA. The positively charged outer layer of the TAT peptide ensures cell membrane penetration and a nanoparticle size (<60 nm) that matches the nuclear pore complex (~70 nm), but promotes escape in the acidic environment of the lysosome, subsequently entering the nucleus and cytoplasm. The ROS generated by SDT cleaves the Nrf2 pathway (downregulating Nrf2 protein expression), while TIR@siRNA directly damages DNA in the nucleus, activates the mitochondrial apoptosis pathway, and induces ICD. In the CT26 tumor model, this synergistic effect led to a significant upregulation of ICD markers such as CRT/HMGB1/ATP, a significant increase in DC maturation rate, a significant increase in CD8^+^T cell infiltration, and a decrease in the proportion of immunosuppressive cells such as Treg/MDSC. Combined with DPPPA-1 anti-PD-L1 peptide therapy, the primary tumor achieved complete regression, intestinal metastasis was significantly inhibited, and tumor re-challenge experiments showed long-term immune memory protection, confirming the powerful synergistic effect of SDT-gene-immune combination (151).

#### 5.2.3 Integration of gene therapy

**Figure 9:**
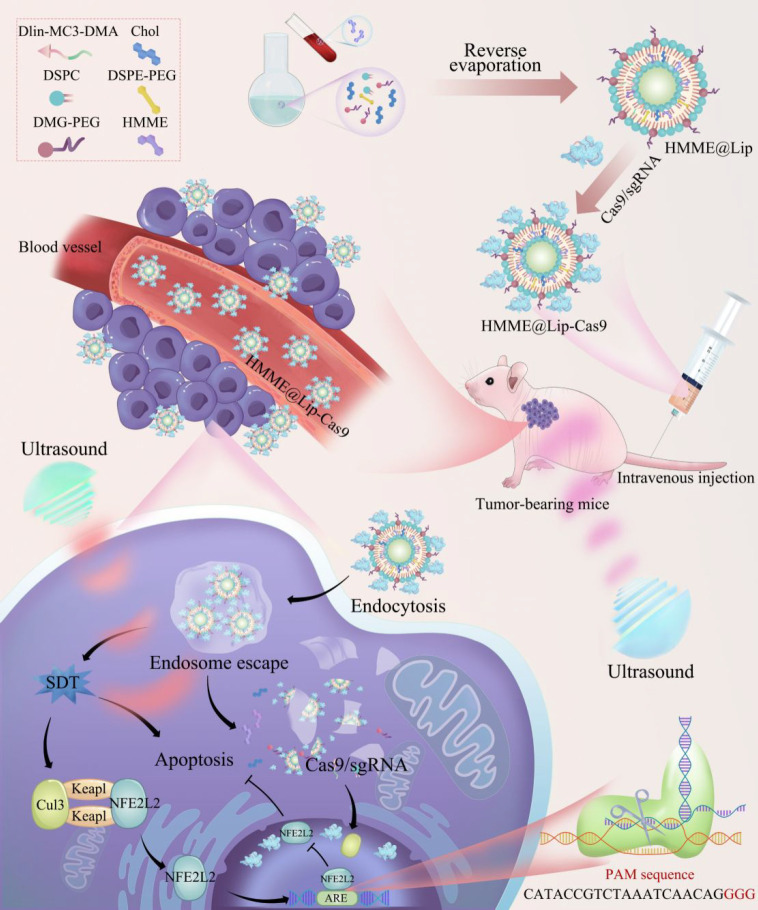
Schematic illustration of the designed strategy of the US-mediated CRISPR/Cas9 delivery system to enhance tumor SDT performance by amplifying oxidative stress. Preparation of HMME@Lip-Cas9 nanosystem and US-controlled CRISPR/Cas9 knock down target genes (NFE2L2). Reproduced from Yin H, Sun L, Pu Y, et al. Ultrasound-Controlled CRISPR/Cas9 System Augments Sonodynamic Therapy of Hepatocellular Carcinoma. ACS Cent Sci. 2021;7(12):2049-2062. doi:10.1021/acscentsci.1c01143. ^©^ The author(s). Creative Commons Attribution License.

The strategy of integrating gene therapy with SDT-ICD involves silencing or editing drug resistance-related genes (such as hypoxia-inducible factor HIF-1α or PD-L1) in TME using small interfering RNA (siRNA) or CRISPR-Cas9 systems. This enhances the sensitivity of tumor cells to SDT-ICD and reduces angiogenesis and immune escape. This strategy often employs cationic carriers or responsive nanosystems to achieve intracellular delivery and protection of nucleic acids, avoiding rapid degradation (152). For example, the siHIF-1α@SDT nanosystem uses cationic liposomes loaded with siHIF-1α (siRNA targeting HIF-1α) and IR780 (a near-infrared acoustic sensitizer). The ultrasonic cavitation effect promotes the endocytosis of nanoparticles and the release of nucleic acids, successfully silencing HIF-1α mRNA expression (reducing it by 82%), thereby blocking the hypoxia-resistant pathway and reducing the expression of vascular endothelial growth factor (VEGF) and PD-L1 (VEGF promotes tumor angiogenesis, and PD-L1 promotes immunosuppression). In the KPC pancreatic cancer model, this synergy reduced angiogenesis by 70%, increased CD8^+^ T cell infiltration by 6.2 times, and, in combination with SDT-ICB, reduced the volume of pancreatic tumors *in situ* by 96% and extended survival by more than 100 days, highlighting the potential of gene silencing in overcoming the high hypoxia and fibrotic TME of pancreatic cancer (153). In addition, as shown in **Figure 9**, the CRISPR-Cas9-targeted PD-L1 SDT platform has further realized the application at the gene editing level: by delivering Cas9 protein and PD-L1-targeting guide RNA through nanocarriers, the PD-L1 gene knockout rate exceeded 75%, directly blocking checkpoint signals at the gene level, avoiding the drug resistance and high cost problems of traditional antibody therapy. This integrated strategy has shown excellent performance in enhancing SDT-ICD sensitivity and immune activation, providing a new path for future personalized gene-immunotherapy (154).

## 6 Advances, challenges, and trends in clinical research

Clinical research on SDT in cancer immunotherapy has entered a multicenter, randomized controlled phase. The focus at this stage is to validate the safety and efficacy of SDT in refractory tumors such as gliomas, particularly for cancer types where traditional surgery, radiotherapy, or chemotherapy have limited effectiveness. Based on sonodynamic agents such as 5-ALA (a pro-sonic sensitizer that can be converted to protoporphyrin IX in tumor cells, thereby enhancing ROS production), combined with a strategy of magnetic resonance-guided focused ultrasound (MRgFUS, a non-invasive technique that precisely focuses ultrasound waves on brain tumors, avoiding craniotomy), it has shown significant potential to prolong patient survival and improve quality of life in preliminary clinical trials. This method eliminates the risk of surgery and improves the delivery of drugs by transiently opening the blood-brain barrier (BBB) (155). In spite of the advancement we have made, SDT still faces many issues because the ICD induction efficiency, targeted delivery accuracy and TME heterogeneity- these could affect the use of this widely. In the future, SDT is expected to undergo artificial intelligence (AI) optimized design, image-guided integrated technology and personalized nanoplatform, etc. SDT will ultimately achieve an accurate translation from basic laboratory research to clinical application, promoting cancer immunotherapy into a new era (156).

### 6.1 Overview of current clinical trials

Currently, the clinical trials of sound sensitizer therapy are in phases I, II and III, which are meant for safety assessment, early efficacy verification and efficacy verification in large populations respectively. Most trials are on CNS (central nerves system) tumours like gliomas (157). More details about this therapy include useful information about 5-ALA which is the typical sonosensitiser. The benefit of this sonosensitizer is its accumulation in tumor tissue to generate ultrasound-activated ROS-induced ICD (158). SDT is combined with MRgFUS or NaviFUS systems (a type of guided focused ultrasound device) in clinical trials, allowing ultrasound activation of SDT to be accurately targeted at the tumor area while sparing other normal tissues (159). Clinical trials are done on the SDT for monotherapy as well as their combination with chemotherapy or immunotherapy. This improves the overall survival and quality of life (160).

Another phase I/II trial was NCT05362409, which focused on validating the dose-escalation safety of SDT in rGBM. The trial used a gradual dose-escalation regimen, with the highest dose group (5-ALA 20 mg/kg) showing a PFS of 5.5 months and an incidence of grade 3 or higher adverse events (AEs, such as serious toxic side effects) of less than 8%. The significant side effects comprised reversible photosensitivity and headache typically resolved after a few days. Results of this trial provided evidence for dose optimization and safety of subsequent higher phase trials (161). Furthermore, a phase II study (NCT05123534) used SDT combined with MRgFUS-guided focused ultrasound to activate the ALA metabolite protoporphyrin IX, thereby promoting tumor cell death and prolonging survival in a glioma model. Results showed a median survival of 9–12 months in patients with gliomas. Preclinical studies have indicated that SDT has a protective effect on neurological function (162).

In China, in the ChiCTR2200065992 study, a phase I clinical trial (enrolling 9 patients), patients with recurrent glioblastoma (rGBM) received SDT combined with temozolomide (TMZ, the standard chemotherapy drug for GBM). SDT uses a hematoporphyrin derivative (hyporfin) as a sonosensitive agent combined with low-frequency ultrasound activation. After treatment with the TMZ-enhanced regimen, no significant hematologic or hepatorenal toxicity related to SDT was observed in the patients. The main adverse event was myelosuppression caused by TMZ. Preliminary results showed that one patient maintained stable disease (SD) for 155 days, the median progression-free survival for patients with progression was 84 days, and the median overall survival after recurrence was 202.5 days. Therefore, SDT combined with TMZ showed good safety and potential synergistic effect in the treatment of rGBM (163). Another practical example is the phase I clinical trial (later expanded to phase IIa, combining SDT with radiotherapy (RT)) for brainstem gliomas. This study used a hematoporphyrin derivative as a sonosensitive agent, combined with low-frequency ultrasound to activate SDT, administered concurrently with RT, to assess safety, tumor control rate, and survival benefit. Magnetic resonance imaging was performed during the trial to evaluate the tumor, and adverse events were recorded. All adverse events were grade 1 to 2; no grade 3 or more severe adverse events were observed. Treatment was well tolerated, and no dose-limiting toxicities were observed. There were no treatment-related deaths during the treatment. Preliminary results show that SDT+RT/chemotherapy-radiotherapy can significantly improve the disease control rate of brainstem gliomas and is well tolerated, providing a new combination therapy strategy for refractory brainstem tumors (164).

### 6.2 Challenge analysis

Although clinical trials initially validated the safety and efficacy of SDT, it still faces numerous bottlenecks caused by technical, biological, and clinical challenges. If these issues are not resolved, they may hinder SDT from entering Phase III clinical trials and its eventual approval process. Some of the main issues will be discussed in detail below. ① Low ICD induction efficiency: Clinical data show that less than 45% of patients reach the threshold expression levels of DAMPs (CRT exposure >70%, HMGB1 release >300 ng/mL), which is mainly attributed to the tumor hypoxic environment (oxygen partial pressure pO_2_ <10 mmHg) and high glutathione levels (GSH >5 mM, leading to antioxidant effects from reactive oxygen species). Therefore, immune activation is insufficient to trigger a systemic antitumor response (165). When sonosensitive agents have poor targeting and low stability, their brain retention time is less than 24 hours, blood-brain barrier penetration rate is less than 15%, and serum half-life is about 45 minutes, making it difficult to maintain effective concentrations. Repeated administration due to reduced efficacy can lead to cumulative toxicity and may even be fatal (166). TME is heterogeneous, with TMB ranging from 2 to 50 mutations/Mb due to clonal evolution. Treatment response differs by more than 5-fold between “cold tumors” (poor immune cell infiltration) and “hot tumors” (hyperimmune activity). Therefore, the efficacy of SDT varies from patient to patient, making standardization of treatment regimens challenging (167). When SDT is used in combination with ICB, the risk of immune-related adverse events (such as colitis, pneumonia, and other serious immune responses) increases due to T cell overactivation. Cytokine storms or organ function must be regularly assessed through clinical monitoring to ensure that the efficacy risk is within a reasonable range (168). HMGB1 protein acts as both an immunostimulant and a component of DAMP molecules that promotes cancer cell ablation. However, if its concentration exceeds 600 ng/mL, this DAMP protein can stimulate the receptor RAGE, promoting tumor angiogenesis and metastasis. This double-edged sword phenomenon necessitates the development of strategies to capture or inhibit HMGB1 activity in order to maximize immune activation while minimizing negative effects (169). The lack of consistency in parameters (e.g., ultrasound intensity of 0.5–3 W/cm², irradiation duration of 5–30 minutes) leads to poor comparability between trials, hindering the progress of large-scale randomized controlled trials (170). These difficulties indicate that multi-party collaboration is needed to optimize SDT protocols (171).

## 7 Conclusions

Stimulus-responsive sonodynamic immunotherapy precisely responds to signals in the tumor microenvironment, achieving targeted delivery and controllable activation of sonosensitive agents. Under ultrasound, it effectively induces immunogenic cell death, remodels the immunosuppressive microenvironment, and thus activates a systemic anti-tumor immune response. This strategy significantly overcomes the limitations of traditional therapies and synergistically combines with immune checkpoint blockade, chemotherapy, and other methods to achieve higher tumor clearance rates and more durable immune memory. Although challenges remain regarding reactive oxygen species generation efficiency, clinical translation, and nanocarrier stability, the rapid development of multifunctional nanoplatforms and biodegradable materials provides new pathways for developing low-toxicity, precise, and personalized cancer treatments, with broad clinical application prospects.
